# Discovery of a New Rosamicin Derivative from Endophytic *Micromonospora rosaria* FoRo54 Using Genome Mining Technology

**DOI:** 10.3390/molecules31020301

**Published:** 2026-01-14

**Authors:** Zhi-Bin Zhang, Qi Liu, Guo-Dong Song, Yi-Wen Xiao, Ri-Ming Yan, Du Zhu

**Affiliations:** 1 Key Laboratory of Biodiversity Conservation and Bioresource Utilization of Jiangxi Province, College of Life Science, Jiangxi Normal University, Nanchang 330022, China; zzbbio@jxnu.edu.cn (Z.-B.Z.); 15270610127@163.com (Q.L.); songgd2079@163.com (G.-D.S.); rimingyan@163.com (R.-M.Y.); 2 Key Laboratory of Natural Microbial Medicine Research of Jiangxi Province, College of Life Science, Jiangxi Science and Technology Normal University, Nanchang 330013, China; xyw1152858687@163.com

**Keywords:** *Micromonospora rosaria*, antibacterial activity, genome mining, macrolides

## Abstract

Endophytic FoRo54 was isolated from the roots of *Oryza rufipogon* (Dongxiang wild rice) collected in China. Based on morphological characteristics and phylogenetic analysis of the 16S rRNA gene sequence, strain FoRo54 was identified as closely related to *Micromonospora rosaria*. The complete genome of FoRo54 consists of a linear chromosome of 7,057,852 bp with a GC content of 73.8 mol%. Genome mining using antiSMASH revealed 27 biosynthetic gene clusters (BGCs) potentially involved in secondary metabolite biosynthesis, including those associated with kanamycin, rosamicin, and asukamycin, consistent with the antibacterial activities of the strain. Application of a combined genome mining strategy enabled further exploration of the strain’s metabolic potential. One new rosamicin derivative, *N*-demethyl rosamicin (**1**), together with three known compounds, rosamicin (**2**), SCH 23831 (**3**), and tylactone (**4**), were isolated from fermentation broth. Antibacterial evaluation revealed that compounds **1**-**4** exhibited potent inhibitory activity against *Staphylococcus aureus*. Furthermore, based on genomic analysis, the biosynthetic pathway and putative gene functions responsible for these metabolites were proposed. Collectively, these findings highlight the metabolic versatility of the endophytic *Micromonospora rosaria* FoRo54, underscoring its potential as a valuable source of novel bioactive metabolites and providing a genomic framework for future heterologous expression and functional genetic characterization.

## 1. Introduction

Actinomycetes are the main producers of natural bioactive compounds, accounting for nearly 45% of more than 20,000 microbial metabolites reported to date, and they play pivotal roles in medicine, industry, and agriculture [1,2]. However, as research advances, the possibility of isolating novel metabolites from conventional actinomycete sources has progressively diminished [3,4]. Many compounds discovered from these well-studied environments are already known, and structurally new scaffolds have become increasingly rare. Meanwhile, existing bioactive compounds cannot fully meet the demands of practical applications. As a result, attention has shifted toward rare actinomycetes that inhabit unique or underexplored ecological niches [5,6].

*Micromonospora*, a large group of rare actinomycetes, have produced a remarkable array of structurally complex and biologically active metabolites with potential for natural product discovery and pharmaceutical development [7,8]. Among these, macrolides have attracted sustained pharmaceutical interest due to their diverse activities. They are structurally defined by a 14- to 16-membered lactone ring functionalized with sugar moieties via glycosidic bonds [9]. Rosamicin is a 16-membered macrolide antibiotic produced by *Micromonospora rosaria* [10]. It contains a branched lactone and deoxyhexose sugar D-desosamine at the C-5 position, which remains clinically important for treating certain Gram-positive infections [11]. However, discovering novel antibacterial macrolides through traditional approaches has become increasingly challenging. This challenge has shifted focus towards genomic strategies. Comparative analyses reveal that actinomycetes like *Micromonospora* spp. harbour numerous silent biosynthetic gene clusters [12]. Systematically activating these cryptic pathways now represents a promising frontier for uncovering novel chemical diversity and revitalizing natural product discovery for drug development [13,14].

Previous bioactivity screens have revealed that Dongxiang wild rice hosts a rich diversity of endophytic microorganisms [15,16]. Among them, endophytic FoRo54, isolated from the roots, exhibited potent antibacterial activity [17]. To further explore its metabolic potential, this study employed a multi-culture combinatorial fermentation strategy to activate the production of cryptic secondary metabolites. The bioactive compounds obtained were subsequently analyzed using bioinformatics tools to identify the associated biosynthetic gene clusters and predict their biosynthetic pathways. These findings provide a valuable foundation for the discovery of novel bioactive molecules and functional characterization of the genes involved in their biosynthesis.

## 2. Results and Discussion

### 2.1. Identification of Strain FoRo54

Strain FoRo54 was grown on Gause’s agar medium for 14 days, producing dark green colonies with a dry, aerial mycelia (Figure S1A). Strain FoRo54 showed well-developed intracellular mycelium, with both branched and unbranched spores observed under a scanning electron microscope. Some sporophores carried single spores with rough, protuberant surfaces (Figure S1B). The spores, approximately 0.5~0.8 μm in diameter, exhibited the characteristic morphological features of the genus *Micromonospora*.

The 16S rRNA gene sequence of strain FoRo54 (GenBank accession no. KM370076) was obtained and analyzed using the EzTaxon database. The sequence exhibited the highest similarity to *Micromonospora rosaria* DSM803^T^, sharing a sequence similarity of 99.83%. Phylogenetic analysis (Figure S1C) further supported this relationship, with strain FoRo54 forming a well-supported clade with the type strain DSM 803^T^. Taken together with its morphological characteristics, these results indicate that strain FoRo54 belongs to the species *Micromonospora rosaria*.

### 2.2. Antibacterial Activity of Strain FoRo54

In this study, four pathogen bacteria, *S. aureus*, *E. coli*, *B. subtilis,* and *X. oryzae* pv. *Oryzicola*, were chosen for antimicrobial activity screening. As shown in Figure S2, the fermentation broth of strain FoRo54 exhibited pronounced antibacterial activity against all tested microorganisms. The most potent inhibition was observed against *S. aureus* and *E. coli*, with an inhibition zone measuring approximately 20 mm in diameter. Moderate inhibitory effects were also noted against *B. subtilis* and *X. oryzae* pv. *oryzicola*. Collectively, these findings demonstrate that strain FoRo54 produces metabolites with broad-spectrum antibacterial activity which might have the capacity to produce different types of active antibiotics.

### 2.3. Genome Sequencing and Bioinformatic Analysis of Strain FoRo54

**Figure 1:**
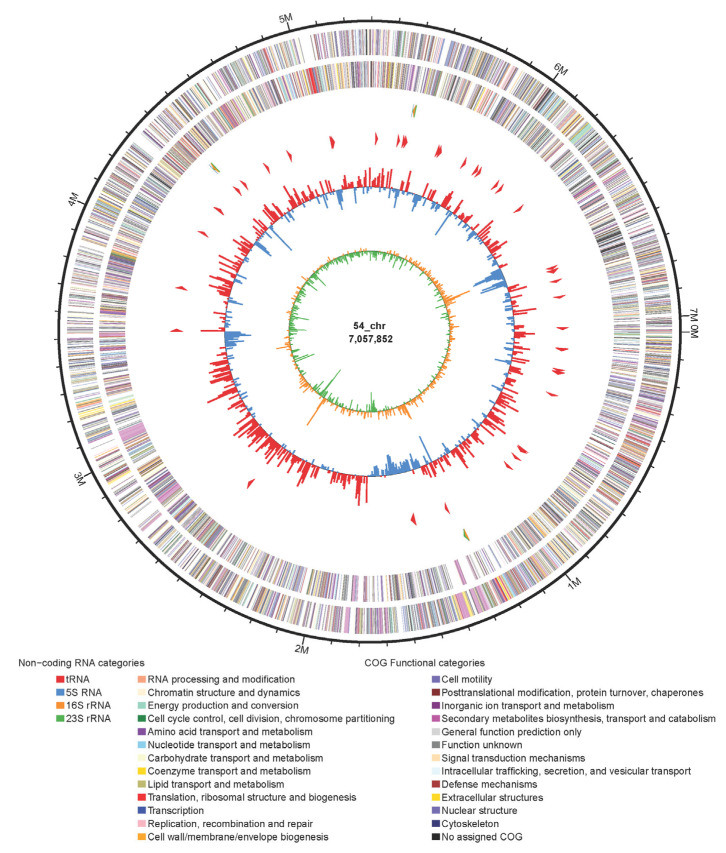
The complete genome of strain FoRo54.

The complete genome sequence of strain FoRo54 was obtained by using Illumina Hiseq and Nanopore platform. The complete genome consists of a 7,057,852 bp circular chromosome with a high G + C content of 73.8% (Table 1 and Figure 1). The chromosome genome was predicted to contain 6128 ORFs, including 53 tRNA genes and 9 rRNA genes. Among the identified genes, 4887 and 939 genes were classified into functional categories based on clusters of orthologous genes of proteins (COG) and GO designations, respectively (Table 1). A total of 2714 genes were assigned to KEGG pathways. A total of 110 genes associated with antibiotic resistance were identified, which may support the strain’s capacity to produce and tolerate its own antibiotic metabolites. A total of 403 genes were assigned to be carbohydrate-active enzymes.

Based on the genome mining results, up to 27 putative biosynthetic gene clusters were found (Table S1), revealing that this bacterium has the potential to produce a wealth of secondary metabolites. Among these clusters, six encode nonribosomal peptide synthetase (NRPS), five encode hybrid PKS-NRPS, six encode polyketide synthase (PKS), two are responsible for terpene biosynthesis, and seven blong to other biosynthetic types.

Comparative genomic analysis against characterized natural product BGCs revealed that over half of the clusters exhibited less than 30% sequence similarity to known counterparts.

Remarkably, three clusters lacked detectable homology to any previously reported biosynthetic gene clusters. These findings suggest that strain FoRo54 possesses substantial genetic potential for the biosynthesis of novel natural products, underscoring its promise as a source of new bioactive compounds.

### 2.4. Isolation and Structural Elucidation of Metabolites

**Figure 2:**
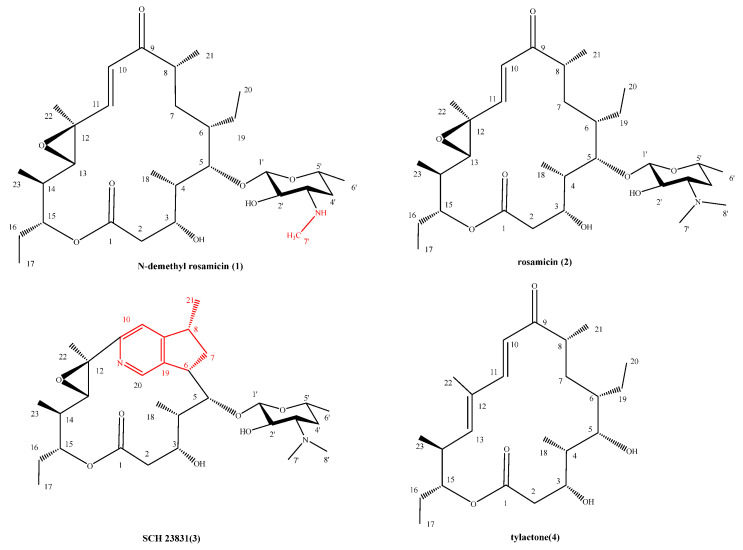
Chemical structures of compounds **1**-**4**.

With the guidance of the genome mining results, strain FoRo54 was fermented using OSMAC approach, within which Z2 fermentation showed strong antibacterial activity [17]. This activity was then used to guide the chromatographic fractionation, leading to the isolation of the following active compounds: *N*-demethyl rosamicin (**1**), together with three known compounds rosamicin (**2**), SCH 23831 (**3**), and tylactone (**4**) (Figure 2). Their structures were established by NMR spectroscopic analyses.

**Figure 3:**
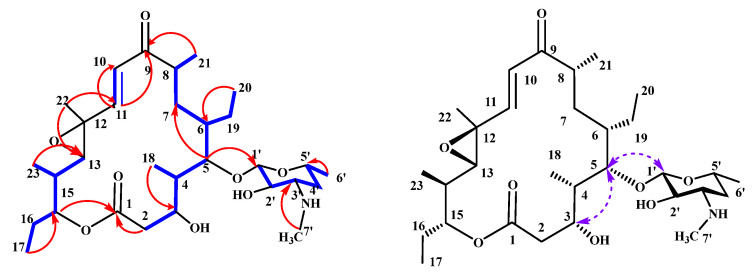
The key H-^1^H COSY (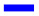), HMBC (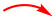), and NOESY (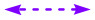) correlations in **1**.

Compound **1** was obtained as a white amorphous powder. Its molecular formula was determined to be C_30_H_51_NO_8_, as established from a HR-ESI-MS ion peak at *m*/*z* 554.3707 [M + H]^+^ (calcd. for C_30_H_52_NO_8_, 554.3687, *Δ*ppm 3.6) indicating six degrees of unsaturation. The ^1^H NMR data (Table 2) revealed **1** had two olefinic protons at *δ*_H_ 6.43, 6.70 (d, *J* = 15.7 Hz); one anomeric proton at *δ*_H_ 4.31 (d, *J* = 6.9 Hz); and eight methyl groups at *δ*_H_ 2.68 (s), 1.47 (s), 1.26 (d, *J* = 6.2 Hz), 1.17 (d, *J* = 6.9 Hz), 1.12 (d, *J* = 6.7 Hz), 1.10 (d, *J* = 6.9 Hz), 0.89 (t, *J* = 7.4 Hz), and 0.86 (t, *J* = 7.2 Hz), respectively. The ^13^C and HSQC spectra (Table 2, Figures S5 and S6) indicated that 30 carbons were assigned as two carbonyl groups (*δ*_C_ 204.0, 174.4); two olefinic sp^2^ carbons (*δ*_C_ 152.1, 124.8); one epoxygenated sp^3^ quaternary carbon (*δ*_C_ 61.2); seventeen sp^3^ methine carbons, including one anomeric methine carbon (*δ*_C_ 104.6), six oxygenated sp^3^ methine carbons (*δ*_C_ 80.7, 78.0, 72.7, 69.6, 69.3), and five sp^3^ methylenes carbons; and eight methyl carbons including one N-methyl (*δ*_C_ 30.9). Analysis of the ^1^H-^1^H COSY spectrum of **1** (Figure S7) revealed the presence of five spin systems (bold in Figure 3). Key HMBC correlations from H_2_-2 (*δ*_H_ 2.62, 2.26) and H-15 (*δ*_H_ 4.86) to C-1 (*δ*_C_ 174.4), along with from H-8 (*δ*_H_ 2.65) and H-11 (*δ*_H_ 6.70) to C-9 (*δ*_C_ 204.0), and from H-11 (*δ*_H_ 6.70) and H-13 (*δ*_H_ 2.83) to C-12 (*δ*_C_ 61.2), defined a 16-membered macrolactone ring. The presence of one monosaccharide unit was suggested by the existence of an anomeric proton signal (*δ*_H_ 4.31). Through analysis of the remaining NMR data of **1**, one glycosyl moiety comprising C-1′ to C-6′ was identified to be D-desosamine. HMBC correlation from H-1′ (*δ*_H_ 4.31) to C-5 (*δ*_c_ 80.7) demonstrated the desosamine was O-linked to C-5, which is common among 16-membered macrolides [18].

Comparative analysis of the NMR data for compound **1** and the co-isolated compound **2** (rosamicin) (Table 2) indicated that compound **1** possesses a 16-membered macrolide similar to rosamicin [19] but lacks the methyl signal of H_3_-8′. This observation, together with a molecular weight difference of 14 Da, revealed that compound **1** contains a demethylated desosamine. This inference was corroborated by the upfield shift of the H_3_-7′ (*δ*_H_ 2.33 to 2.68) and downfield shift of the C-3′signal (from *δ*_C_ 65.9 to 61.0) in compound **1** compared to rosamicin. Thus, on the basis of the spectroscopic and MS data, compound **1** was identified as a new demethylated analogue of rosamicin, designated *N*-demethyl rosamicin.

The relative stereochemistry of compound **1** was elucidated by NOESY experiments (Figure 3 and Figure S9) and values of ^1^H-^1^H coupling (Table 2). NOESY correlations were observed between H-3/H-5, and together with the large coupling constant of H-4 and H-5 (*J*_H-4, H-5_ = 9.8 Hz) they established the relative configuration of C-3 to C-6 as 3R*/4S*/5S*/6S*. The geometrical configuration of the double-bond moiety C-10/C-11 was deduced as E based on the large coupling constant of H-10/H-11 (*J*_10, 11_ = 15.7 Hz). The anomeric proton of the desosamine moiety (H-1′, *δ*_H_ 4.31, d, *J* = 6.9 Hz), together with the NOESY correlation, was observed between H-5/H-1′, which confirmed a β-glycosidic linkage. Given that natural desosamine is exclusively reported in the D-form [11,19], the relative configuration of 1 is proposed to be consistent with that of rosamicin.

**Figure 4:**
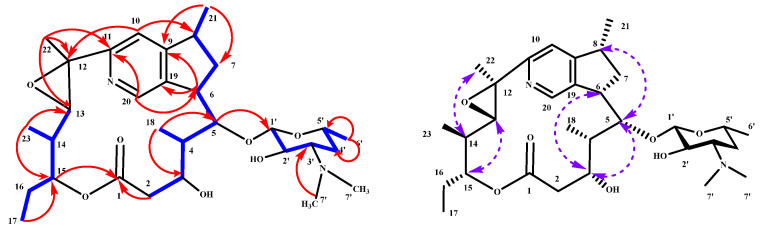
The key H-^1^H COSY (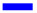), HMBC (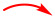), and NOESY (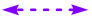) correlations in **3**.

Compound **3** was obtained as a white amorphous powder, soluble in methanol. Its molecular formula was determined to be C_31_H_48_N_2_O_7_, as established from a HR-ESI-MS ion peak at *m*/*z* 561.3578 [M + H]^+^ (calcd. for C_31_H_49_N_2_O_7_, 561.3540, *Δ*ppm 6.7) indicating nine degrees of unsaturation. Analysis of the ^1^H and ^13^C NMR data revealed eight methyl, four methylene, twelve methine, two aromatic, and five quaternary carbons (including one carbonyl carbon at *δ*_C_ 174.8) (Table S3, Figures S13–S15). The ^1^H-^1^H COSY spectrum of **3** indicated the presence of three spin systems (Figure 4 and Figure S16). The first spin system comprising C-2 to C-8 was identified from consecutive COSY correlations from methylene protons H_2_-2 (*δ*_H_ 2.20, 1.57) to H-8 (*δ*_H_ 1.28). The second spin system comprising C-13 to C-17 was constructed based on an array of COSY correlations from the oxygenated methine H-13 (*δ*_H_ 2.10) to H-17 (*δ*_H_ 0.88). Three branched methyl groups, H_3_-18 (*δ*_H_ 0.98), H_3_-21 (*δ*_H_ 1.28), and H_3_-23 (*δ*_H_ 1.12), were, respectively, assigned to C-4, C-8, and C-14 by their COSY correlations with the corresponding methine protons. Key HMBC correlations from H_2_-2 (*δ*_H_ 2.20, 1.57) and H-15 (*δ*_H_ 4.72) to C-1 (*δ*_C_ 174.4), along with from H-21 (*δ*_H_ 1.28) to C-7 (*δ*_C_ 41.5) and C-9 (*δ*_C_ 161.9), from H-10 (*δ*_H_ 7.07) to C-9 (*δ*_C_ 161.9) and C-12 (*δ*_C_ 63.5), and from H-22 (*δ*_H_ 1.80) to C-11 (*δ*_C_ 163.7) and C-13 (*δ*_C_ 73.6), defined a 16-membered macrolactone ring as part of the planar structure (Figure 4 and Figure S17). Additional HMBC correlations from H-6 (*δ*_H_ 3.24) to C-9 (*δ*_C_ 161.9) and C-19 (*δ*_C_ 139.7), and from H-20 (*δ*_H_ 8.33) to C-9 (*δ*_C_ 161.9), C-11 (*δ*_C_ 163.7), and C-19 (*δ*_C_ 139.7), along with from H-10 (*δ*_H_ 8.33) to C-8 (*δ*_C_ 37.8) and C-11 (*δ*_C_ 163.7), indicated the presence of a nine-membered pyridine ring. The planar structure of **3** is consistent with that of SCH 23831 [20]. Although compound **3** has been previously reported, detailed 2D NMR spectroscopic data have not been described. In this study, the partial relative stereochemistry of compound **3** was elucidated by NOESY experiments (Figure 4 and Figure S18). NOESY correlations were observed between H-3/H-5, H-3/H-6, and H-5/H-8, combing with the H-13/H-15 and H_3_-22/H-14 established the relative configurations of C-3 to C-6 as 3R*/4S*/5S*/6S*/8R* and C-12 to C-15 as 12S*/13S*/14R*/15R*. It is biogenetically highly plausible that the relative configurations of compound **3** are consistent with those of known rosamicin members.

Compound **4** was obtained as a white amorphous powder. Its molecular formula was C_23_H_38_O_5_ and it indicated five degrees of unsaturation. Analysis of the ^1^H and ^13^C NMR data revealed six methyl, four methylene, ten methine (including three olefinic), and three quaternary carbons (including one carbonyl carbon) (Table S3). Comprehensive analysis of the 1D and 2D NMR data enabled complete assignment of all proton and carbon resonances (Figures S19–S25). Through comparison with literature data for known macrolactones, compound **4** was identified as tylactone [21].

### 2.5. Propose Biosynthetic Pathway of Rosamicin-Type Compounds

**Figure 5:**
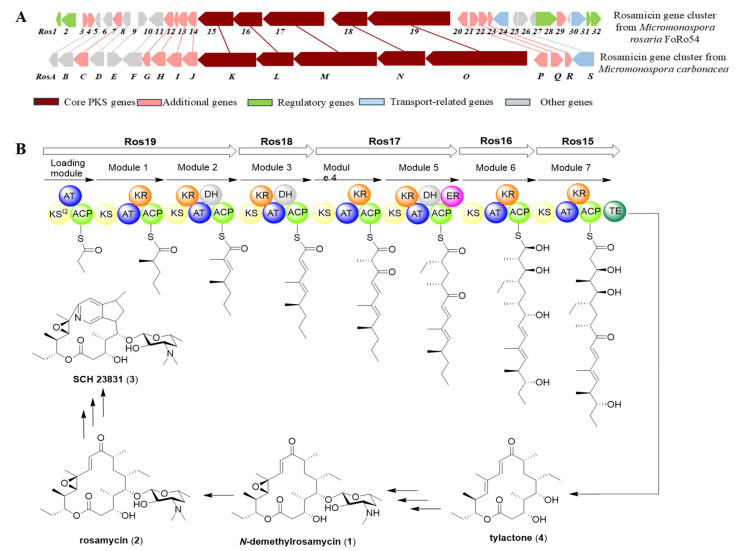
(**A**) The comparison of Ros gene clusters from *M. rosaria* FoRo54 with other rosamicin biosynthetic gene clusters. The homologous genes were linked with lines. (**B**) The proposed biosynthetic pathways of compounds **1-4**.

The genome sequence of strain FoRo54 was analyzed using antiSMASH 8.0. By comparing with the reported biosynthetic gene clusters of rosamicins in the literature [22,23], region 12 was identified as being potentially involved in rosamicin synthesis. Further in-depth analysis of this region using 2ndFind and BLAST software(version 2.16.0) revealed a 77,196 bp DNA sequence (the Ros gene cluster) containing 32 open reading frames, which was determined to be responsible for rosamicin biosynthesis in strain FoRo54 (Table S2). Within this cluster, Ros15-19 encode a modular polyketide synthase (PKS) assembly line, comprising eight modules (including a loading module), which directs the biosynthesis of the 16-membered macrolactone-scaffold tylactone (**4**). This intermediate serves as the core framework that undergoes a series of post-modifications, ultimately giving rise to compounds **1**–**3** (Figure 5).

### 2.6. Antibacterial Activity Assay of Compounds

The antibacterial activities of compounds **1**-**4** were assessed using the broth dilution method (Table 3). Most of the isolates exhibited inhibitory effects against *S. aureus*. In particular, compounds **1**-**4** demonstrated notable activity against *S*. *aureus*, with minimum inhibitory concentration (MIC) values of 4, 4, 16, and 32 μg/mL, respectively, when using tobramycin as a positive control. Furthermore, compounds **1**-**3** displayed moderate inhibitory activity against *E. coli* and *X. oryzae* pv. *oryzicola*, with MIC values of 64 μg/mL, when compared to streptomycin as the reference antibiotic. The potent antibacterial activities of compounds **1** and **2** are consistent with previously reported data for rosamicin [24], suggesting that the absence of the N-methyl group on the amino sugar moiety does not have a significant impact on the antibacterial activity. However, SCH 23831 (**3**) has weak activity against Gram-positive bacteria, which is also in agreement with prior literature reports on this compound [20]. We propose that this structural elaboration could serve as a potential self-detoxification mechanism for the producing strain.

## 3. Materials and Methods

### 3.1. Strains, Culturing Conditions, and Identification

Strain FoRo54 was isolated in October 2015 from the roots of Dongxiang wild rice collected in Dongxiang County (28°14′ N, 116°30′ E), Jiangxi Province, China, and deposited at the College of Life Science, Jiangxi Normal University. It was cultured on Gause’s agar medium (soluble starch 20 g, sodium chloride 0.5 g, ferrous sulfate 0.01 g, potassium nitrate 1 g, sipotassium hydrogen phosphate 0.5 g, magnesium sulfate 0.5 g, agar 15 g, water 1 L, and pH 7.2–7.4) at 28 °C for 14 days, then morphological properties were observed by using a transmission electron microscope (S-3400N, Hitachi, Tokyo, Japan). Genomic DNA was extracted from strain FoRo54 using a commercial bacterial genomic DNA extraction kit, following the manufacturer’s protocol (Takara Bio, DaLian, China). The 16S rRNA gene was amplified via PCR with the universal bacterial primers 27F (5′-AGAGTTTGATCCTGGCTCAG-3′) and 1492R (5′-GGTTACCTTGTTACGA CTT-3′). Each 25 μL PCR mixture contained 12.5 μL of Taq Buffer Mix, 2 μL of each primer, 1 μL of DNA template, and 7.5 μL of ddH_2_O. The amplification programme consisted of an initial denaturation at 95 °C for 5 min, followed by 34 cycles of denaturation at 95 °C for 30 s, annealing at 55 °C for 30 s, and extension at 72 °C for 90 s, with a final elongation step at 72 °C for 10 min [15].

PCR products were purified using a gel extraction kit and sequenced by Shanghai Sangon Biotech Co., Ltd. (Shanghai, China) The resulting sequences were compared against reference sequences in the EzTaxon database (https://eztaxon-e.ezbiocloud.net/;accessed on 7 July 2021) to determine phylogenetic affiliations. A phylogenetic tree was constructed based on the 16S rRNA gene sequence, using the neighbour-joining method implemented in MEGA-X software(Version 10.2.6) with 1000 bootstrap replicates to assess branch reliability [25].

### 3.2. Genome Sequencing, Annotation, and Analysis

The whole-genome of FoRo54 was sequenced using a combined strategy of third-generation nanopore sequencing and second-generation Illumina PE150 sequencing at Biozeron Technology Co., Ltd. (Shanghai, China). Genome assembly was carried out with Uni Cycler (V0.4.8), and annotation was performed using the NCBI Prokaryotic Genome Annotation Pipeline (PGAP). Predicted protein-coding genes were functionally annotated through comparison with the NR, GO, COG, and KEGG databases [26]. The antibiotic and secondary metabolite production gene clusters were examined using the antiSMASH online platform and further refined with 2ndFind for the precise annotation of secondary metabolite genes. Genes or proteins within clusters of particular interest were subjected to homology analysis using BLASTx and BLASTp searches via the online NCBI BLAST service (https://blast.ncbi.nlm.nih.gov; accessed on 10 October 2025).

### 3.3. Antibacterial Activity of Fermentation Broth

Strain FoRo54 was inoculated into a 250 mL Erlenmeyer flask containing 100 mL of Z2 medium (starch 20 g, yeast extract 4 g, peptone 3 g, potassium nitrate 1 g, sipotassium hydrogen phosphate 0.5 g, magnesium sulfate 0.5 g, sodium chloride 0.5 g, ferrous sulfate 0.01 g, water 1 L, and pH 7.2–7.4) and incubated at 28 °C with shaking at 180 rpm (ZHWY2112, Zhicheng, Yidu, China) for 10 days. Following this, the culture broth was centrifuged (H1850R, Xiangyi Instrument, Xiangtan, China) and the supernatant was extracted with ethyl acetate. The organic phase was evaporated (RV10, IKA, Staufen im Breisgau, Germany) until dry and stored at −4 °C for subsequent assays.

The test pathogenic bacteria were the Gram-positive bacteria *Staphylococcus aureus* (ATCC 29213) and *Bacillus subtilis* (ATCC 7508), as well as the Gram-negative bacteria *Escherichia coli* (ATCC 25922) and *Xanthomonas oryzae* pv. *Oryzicola*. They were cultured in LB broth medium (peptone 10 g, beef extract 5 g, sodium chloride 5 g, water 1 L, and pH 7.2–7.4) at 37 °C for 24 h for the subsequent antibacterial assay. Using a bacterial counting chamber under a microscope, the suspensions were adjusted to a final concentration of approximately 1.0 × 10^6^ CFU/mL [27].

The antibacterial activity of FoRo54 fermentation broth extract against four pathogenic bacteria was assessed using the Oxford cups method. The prepared bacterial suspension was mixed with LB agar (peptone 10 g, beef extract 5 g, sodium chloride 5 g, agar 15 g, water 1 L, and pH 7.2–7.4) at a 1:99 ratio and poured into sterile Petri dishes. After solidification, sterilized Oxford cups were placed on the agar surface in a cross arrangement, and 200 μL of the sterile fermentation extract was added to each cup. The positive control contained chloramphenicol (25 μg/mL), while sterile water served as the negative control. Each treatment was performed in triplicate. The plates were incubated at 37 °C for 24 h, after which inhibition zone diameters were measured using a Vernier calliper and recorded [27].

### 3.4. Fermentation, Extraction, and Isolation of Metabolites

Seed cultures were obtained by inoculation into 50 mL Z2 medium in Erlenmeyer flasks, followed by shaking at 120 rpm at 28 °C for 3 days. Subsequently, the seed inoculum (2%, *v*/*v*) was transferred into a 1000 mL Erlenmeyer flask containing 300 mL of Z2 medium and cultivated at 28 °C with shaking at 160 rpm for 12 days, yielding a total of 80 L of fermentation broth.

The fermentation broth was extracted three times with ethyl acetate. The combined organic layers were concentrated under reduced pressure to afford 13.6 g of crude extract. This extract was subjected to silica gel column with dichloromethane/methanol gradient (100:0–0:100, *v*/*v*) to obtain fractions. Fractions with similar TLC profiles were combined to yield six major fractions (Fr.A-Fr.F). Antibacterial activity assays revealed that Fr.D exhibited the strongest inhibitory activity. This active fraction was subsequently purified by reversed-phase medium-pressure liquid chromatography (Santai Technologies, Changzhou, China) and further refined through semi-preparative high-performance liquid chromatography (Waters 1525, Milford, MA, USA), yielding four compounds: compound **1** (4.4 mg), compound **2** (3.8 mg), compound **3** (7.0 mg), and compound **4** (5.0 mg).

The chemical structures of the isolated compounds were elucidated through comprehensive analysis of NMR and MS data, supported by comparison with literature reports. Each purified compound was dissolved in an appropriate deuterated solvent for a nuclear magnetic resonance assay (AVANCEIII 400 spectrometer Bruker, Berlin, Germany) to acquire structural data, and high-resolution mass data was obtained using an Triple TOF™ 5600+ mass spectrometer (AB Sciex, Framingham, MA, USA). HPLC analysis was performed using a sunfire C18 column (5 μm, 4.6 × 250 mm, Waters, USA). The mobile phase consisted of 0.1% formic acid in water (phase A) and acetonitrile (phase B) at a flow rate of 1.0 mLmin^−1^. Gradient flow elution was as follows: 0–30 min 90–10% A, 10–90% B, and 30 min 100%, then hold until 35 min. Silica gel (200–300 mesh, Qingdao Marine Chemical Group Co., Qingdao, China) was used for column chromatography (CC). Thin-layer chromatography (TLC) was performed on silica gel GF254 plates (Qingdao Haiyang Chemical Co., Ltd., Qingdao, China). All solvents used for CC were of analytical grade from Titan Technology Co., Ltd. (Shanghai, China).

### 3.5. Antibacterial Activity of Compounds

The antibacterial activities of the purified compounds were assessed in vitro against the Gram-positive bacteria *S. aureus* and *B. subtilis*, as well as the Gram-negative bacteria *E. coli* and *X. oryzae* pv. *oryzicola*, using the standard broth microdilution assay in 96-well microplates (SpectraMax M2, Molecular Devices, San Jose, CA, USA) [28].

Briefly, bacterial strains were inoculated into LB medium and incubated at 37 °C for 12 h. The cultures were then harvested and diluted with nutrient broth to achieve a final concentration of 1.0 × 10^6^ CFU/mL. Test compounds were dissolved in DMSO to a stock concentration of 256 µg/mL and serially two-fold diluted to obtain concentrations down to 0.5 µg/mL.

For the assay, 100 µL of each compound dilution was added to designated wells in a 96-well plate, followed by 90 µL of nutrient broth and 10 µL of bacterial suspension, resulting in a final bacterial density of 1.0 × 10^5^ CFU/mL per well. Streptomycin sulfate and tobramycin were used as positive controls, while DMSO served as the negative control.

Plates were incubated at 37 °C for 24 h, after which bacterial growth was quantified by measuring optical density at 600 nm using a microplate reader. All assays were conducted in triplicate, and the minimum inhibitory concentrations (MICs) were determined as the lowest compound concentrations that completely inhibited visible bacterial growth.

## 4. Conclusions

In this study, endophytic FoRo54, isolated from the roots of Dongxiang wild rice (*Oryza rufipogon*), was identified as *Micromonospora rosaria* based on morphological characterization and 16S rRNA gene sequence analysis. Antibacterial assays revealed that FoRo54 exhibited broad-spectrum inhibitory activity. Further whole-genome sequencing and analysis of strain FoRo54 revealed a genome size of 7.06 bp harbouring 27 biosynthetic gene clusters (BGCs). Notable, over half of these clusters shared less than 30% sequence similarity to known clusters, with three being entirely novel. Subsequently, guided by genome mining, a rosamicin-producing BGC was targeted, leading to the isolation of four compounds from fermentation extracts: *N*-demethyl rosamicin (**1**), rosamicin (**2**), SCH 23831 (**3**), and tylactone (**4**). Their structures were elucidated by spectroscopic analysis, and a biosynthetic pathway was proposed based on genomic annotation. Antibacterial evaluation showed that compounds **1**-**4** displayed notable inhibitory activity against *S. aureus*, while compounds **1**-**3** also suppressed the growth of *E. coli* and *X. oryzae* pv. *oryzicola*.

Collectively, this work provides a comprehensive genomic and metabolic profile of *M. rosaria* FoRo54, highlighting its biosynthetic potential, and shows the discovery of a new rosamicin analogue, underscoring the value of integrating genome mining for natural product discovery.

## Figures and Tables

**Table 1:** Genome features of strain FoRo54.

Feature	Chromosome Characteristics
Genomic size (bp)	7,057,852
Number of contigs	59
GC content (%)	73.8%
Protein coding Genes	6128
rRNA genes	9
tRNA genes	53
Genes assigned to GO	939
Genes assigned to COG	4887
Genes assigned to KEGG	2419
Genes assigned to NR	5954
Genes assigned to Swissport	3098
Antibiotic resistance genes	110
Carbohydrate-active enzyme genes	403
Secondary metabolite gene clusters	27

**Table 2:** ^1^H NMR (400 MHz) and ^13^C NMR (100 MHz) data for **1** and **2** in CD_3_OD.

No.	1		2	
	*δ*_C_, Type	*δ*_H_ Mult (*J* in Hz)	*δ*_C_, Type	*δ*_H_ Mult (*J* in Hz)
1	174.4, C		174.4, C	
2	41.3, CH_2_	2.64 dd (17.5, 10.0)	41.3, CH_2_	2.62 dd (17.5, 10.0) 2.26 d (17.5)
		2.25 br d (17.5)		
3	67.8, CH	3.83 br d (10.0)	67.8, CH	3.80 d (10.0)
4	42.3, CH	1.89 m	42.3, CH	1.83–1.89 m
5	80.7, CH	3.77 br d (9.8)	80.2, CH	3.73 d (9.8)
6	40.3, CH	1.19 m	40.4, CH	1.16–1.21 m
7	34.1, CH_2_	1.41 m	34.1, CH_2_	1.42 ddd (15.1, 11.7, 4.1)
		1.83–1.89 m		1.83–1.89 m
8	46.7, CH	2.63–2.69 m	46.7, CH	2.63–2.69 m
9	204.0, C		204.0, C	
10	124.8, CH	6.43 d (15.7)	124.8, CH	6.43 d (15.7)
11	152.1, CH	6.70 d (15.7)	151.9, CH	6.70 d (15.7)
12	61.2, C		61.2, C	
13	69.6, CH	2.83 d (9.7)	69.6, CH	2.83 d (9.7)
14	39.1, CH	1.72–1.78 m	39.1, CH	1.72–1.76 m
15	78.0, CH	4.86 m	77.9, CH	4.86 td (9.7, 2.7)
16	25.6, CH_2_	1.48–1.58 m	25.6, CH_2_	1.49–1.58 m
		1.78–1.87 m		1.79–1.87 m
17	9.5, CH_3_	0.89 t (7.4)	9.5, CH_3_	0.91 t (7.4)
18	9.9, CH_3_	1.10 d (6.9)	10.1, CH_3_	1.10 d (6.9)
19	22.2, CH_2_	1.38–1.44 m	22.2, CH_2_	1.38–1.44 m
		1.60–1.70 m		1.64–1.72 m
20	12.5, CH_3_	0.86 t (7.2)	12.5, CH_3_	0.86 t (7.2)
21	17.8, CH_3_	1.17 d (6.9)	17.8, CH_3_	1.17 d (6.9)
22	15.3, CH_3_	1.47 s	15.3, CH_3_	1.47 s
23	14.6, CH_3_	1.12 d (6.7)	14.6, CH_3_	1.12 d (6.7)
1′	104.6, CH	4.31 d (6.9)	105.7, CH	4.31 d (6.9)
2′	72.7, CH	3.25 m	72.3, CH	3.25 dd (10.2, 7.3)
3′	61.0, CH	3.21–3.08 m	65.9, CH	2.60–2.66 m
4′	35.2, CH_2_	1.20–1.28 m	31.9, CH_2_	1.20–1.28 m
		1.80–1.94 m		1.70–1.76 m
5′	69.3, CH	3.68–3.59 m	70.1, CH	3.52 dtt (12.5, 6.3, 3.11)
6′	21.4, CH_3_	1.26 d (6.2)	21.4, CH_3_	1.20 d (6.2)
7′	30.9, CH_3_	2.68 s	40.9, CH_3_	2.33 s
8′			40.9, CH_3_	2.33 s

**Table 3:** Inhibitory effects of compounds **1-4** on bacteria.

Compounds	MIC (μg/mL)			
	*S. aureus*	*B. subtilis*	*E. coli*	*X. oryzae* pv. *oryzicola*
**1**	4	64	32	64
**2**	4	64	32	64
**3**	16	64	64	64
**4**	32	>128	>128	>128
Streptomycin sulphate	NA	NA	8	64
Tobramycin	1	64	NA	NA

Note: NA signifies no detectable activity under the conditions of this assay.

## Data Availability

The original contributions presented in this study are included in the article. Further inquiries can be directed to the corresponding author(s).

## Supplementary Materials

The following supporting information can be downloaded at: https://www.mdpi.com/article/10.3390/molecules31020301/s1, Figures S1 and S2 are associated with the morphological characters, phylogenetic tree, and antibacterial activity of strain FoRo54; Figures S3–S25 are associated with HR-ESI-MS and 1D and 2D NMR spectra data for compounds **1**-**4**. Tables S1 and S2 are biosynthesis gene clusters and deduced functions of ORFs in the 12# biosynthetic gene cluster. Table S3 is NMR data for compounds **3** and **4**.
