# Supplementary material for: Discovery of a New Rosamicin Derivative from Endophytic Micromonospora rosaria FoRo54 Using Genome Mining Technology
Source: Molecules. 2026 Jan 14;31(2):301. doi: 10.3390/molecules31020301 (PMC12844328; doi:10.3390/molecules31020301)
Supplement: Supplementary file 1 [file molecules-31-00301-s001.zip › molecules-4030150-supplementary.pdf]

Supplementary materials

Discovery of a new rosamicin derivative from endophytic *Micromonospora rosaria* FoRo54 using genome mining technology

Zhi-Bin Zhang[1], Qi Liu [1], Guodong Song[1], Yi-Wen Xiao[1,2], Ri-Ming Yan[1] and Du Zhu *[1,2]

[1]Key Laboratory of Biodiversity Conservation and Bioresource Utilization of Jiangxi Province, College of Life Science, Jiangxi Normal University, Nanchang 330022, China; zzbbio@jxnu.edu.cn (Z.-B. Z.); 3082234875@qq.com (Q. L) ; songgd2079@163.com (G.-D. S.);rimingyan@163.com (R.-M. Y.); zhudu12@163.com (D. Z.)
[2] Key Laboratory of Natural Microbial Medicine Research of Jiangxi Province, Jiangxi Science and Technology Normal University, Nanchang, 330013, China; 1152858687@qq.com (Y.-W. X.)

*****Correspondence: Du Zhu
E-mail: zhudu12@163.com;
Tel.: +86-791-88121934.

Supplementary Materials

Contents

Fig. S1 Morphological characters and phylogenetic tree of Strain FoRo54

Fig. S2 Antibacterial activity assay of secondary metabolite extractions from strain FoRo54 fermentation broth against pathogenic bacteria

Figure S3 HRESIMS spectrum of compound **1** in CD_3OD
Figure S4 ^1H NMR (400 MHz) spectrum of compound **1** in CD_3OD
Figure S5 ^{13}C NMR (100 MHz) spectrum of compound **1** in CD_3OD
Figure S6 HSQC spectrum of compound **1** in CD_3OD
Figure S7 ^1H-^1H COSY spectrum of compound **1** in CD_3OD
Figure S8 HMBC spectrum of compound **1** in CD_3OD
Figure S9 NOESY spectrum of compound **1** in CD_3OD

Figure S10 ^1H NMR (400 MHz) spectrum of compound **2** in CD_3OD
Figure S11 ^{13}C NMR (100 MHz) spectrum of compound **2** in CD_3OD

Figure S12 HRESIMS spectrum of compound **3** in CD_3OD
Figure S13 ^1H NMR (400 MHz) spectrum of compound **3** in CD_3OD
Figure S14 ^{13}C NMR (100 MHz) spectrum of compound **3** in CD_3OD
Figure S15 HSQC spectrum of compound **3** in CD_3OD
Figure S16 ^1H-^1H COSY spectrum of compound **3** in CD_3OD
Figure S17 HMBC spectrum of compound **3** in CD_3OD
Figure S18 NOESY spectrum of compound **3** in CD_3OD

Figure S19 ^1H NMR (400 MHz) spectrum of compound **4** in CD_3OD
Figure S20 ^{13}C NMR (100 MHz) spectrum of compound **4** in CD_3OD
Figure S21 HSQC spectrum of compound **4** in CD_3OD
Figure S22 ^1H-^1H COSY spectrum of compound **4** in CD_3OD
Figure S23 HMBC spectrum of compound **4** in CD_3OD
Figure S24 NOESY spectrum of compound **4** in CD_3OD
Figure S25 ^1H-^1H COSY and key HMBC correlations of compound **4**

Table S1 Biosynthesis gene clusters for secondary metabolites in *Micromonospora* sp. FoRo54 predicted by antiSMASH

Table S2 Deduced functions of ORFs in the $12^{\#}$ biosynthetic gene cluster

Table S3 ^1H NMR (400 MHz) and ^{13}C NMR (100 MHz) data for **3** and **4** in CD_3OD

Fig. S1 Morphological characters and phylogenetic tree of Strain FoRo54

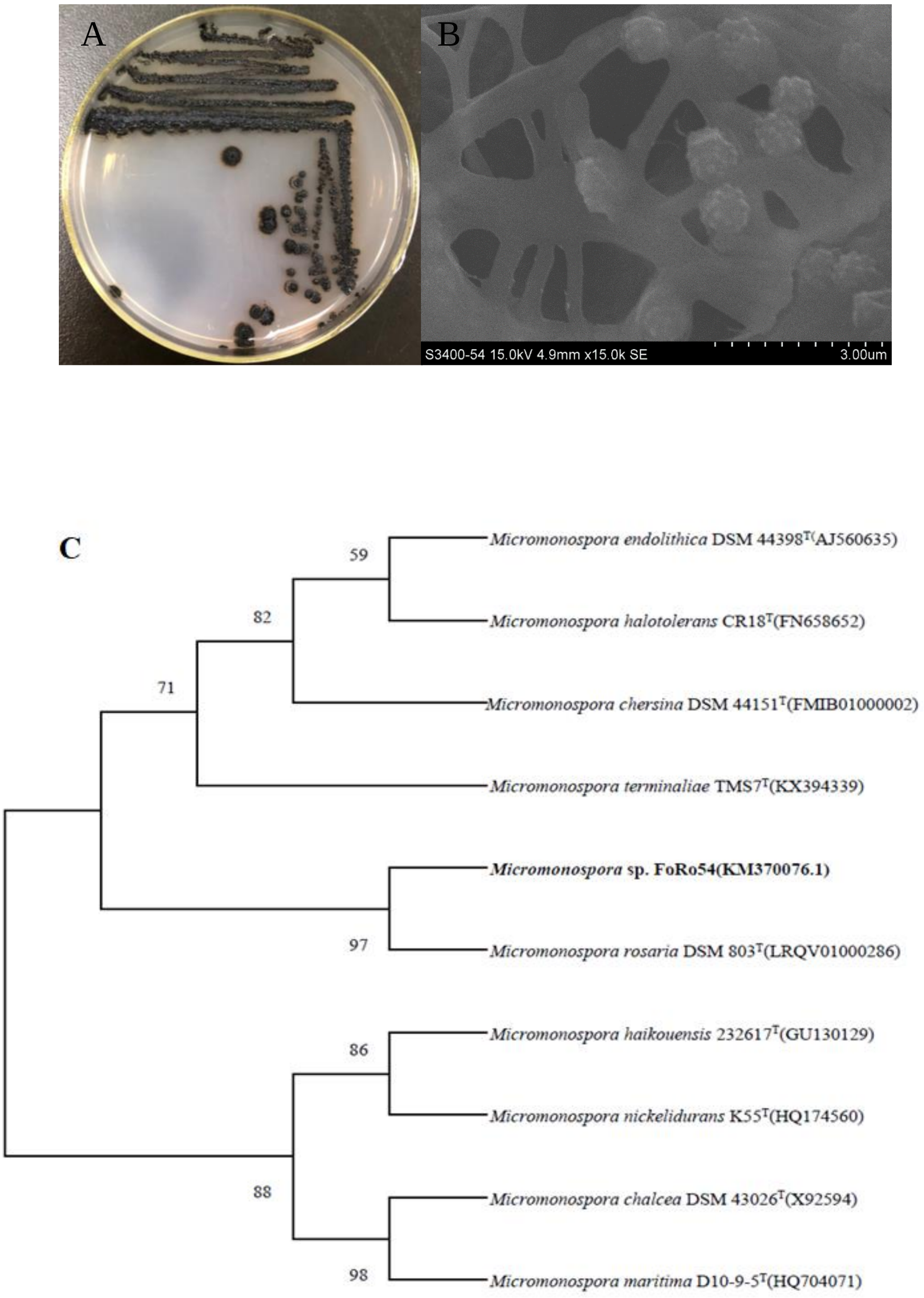

Fig. S2 Antibacterial activity assay of secondary metabolites extractions from strain FoRo54 fermentation broth against pathogenic bacteria

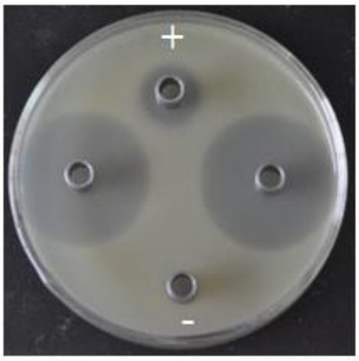

Staphylococcus aureus

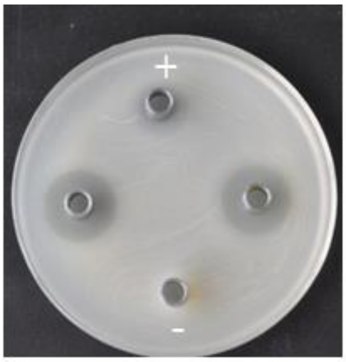

Bacillus subtilis

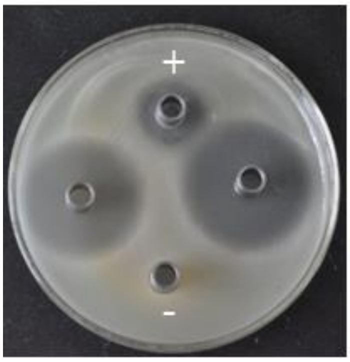

Escherichia coli

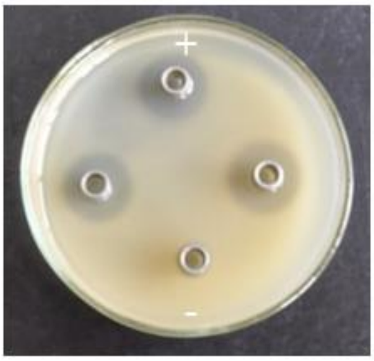

Xanthomonas oryzae pv. *Oryzicola*

Note: Left and right served as the extraction of fermentation broth (1 mg/mL), while positive control (+) contained chloramphenicol (25 μg/mL) and the negative control (-) contained sterile water in petri dish.

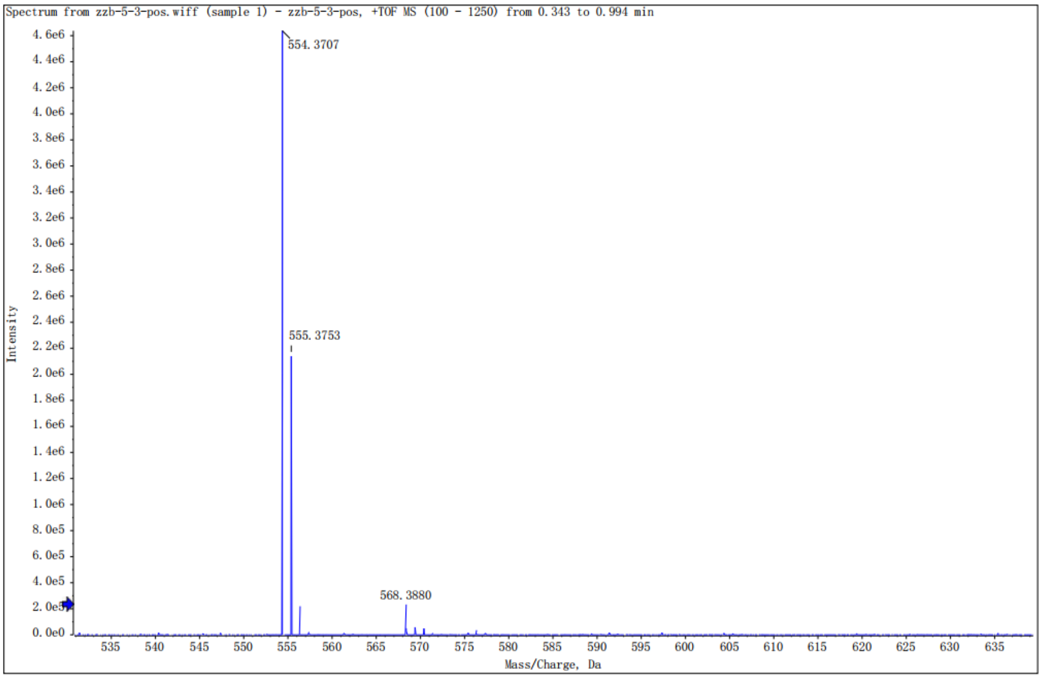

Figure S3 HRESIMS spectrum of compound **1**

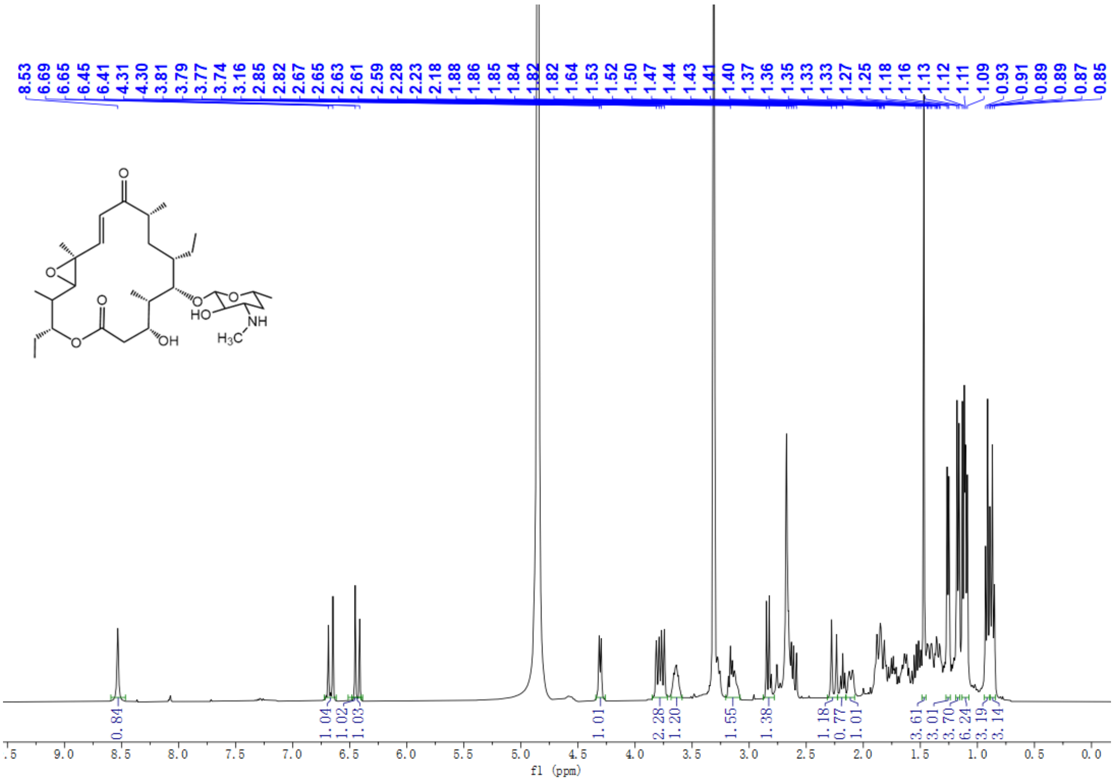

Figure S4 ^{1}H NMR (400 MHz) spectrum of compound **1** in CD_3OD

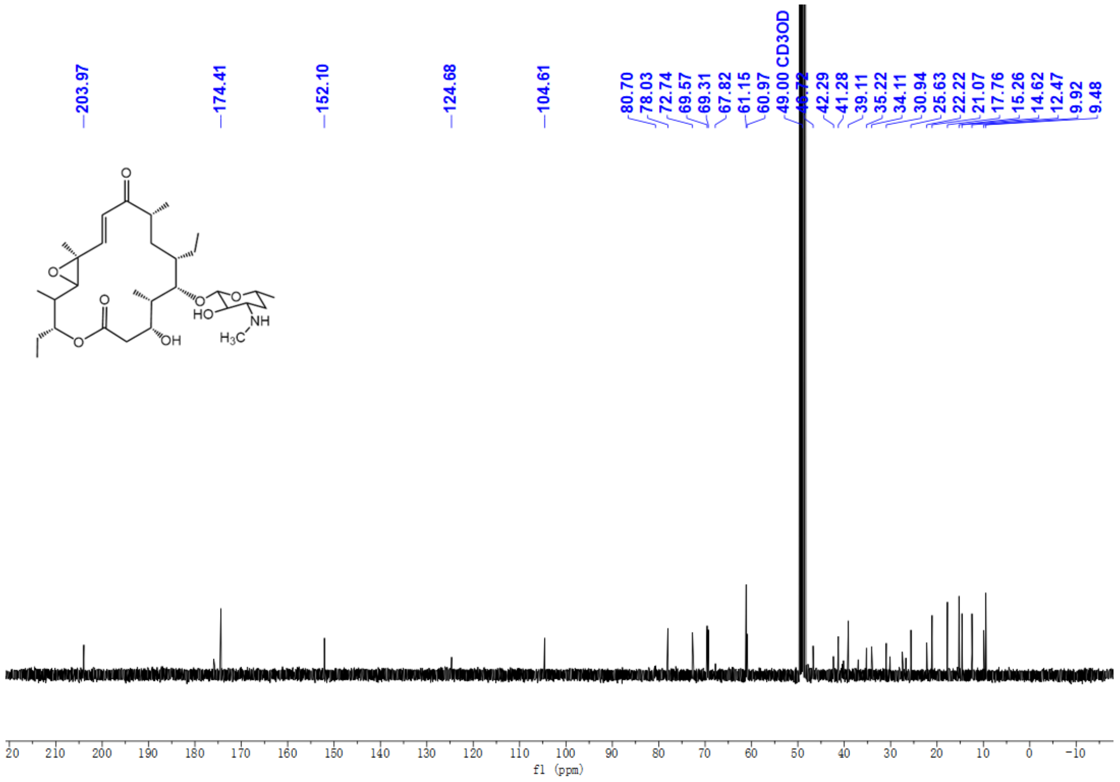

Figure S5 ^{13}C NMR (100 MHz) spectrum of compound **1** in CD_3OD

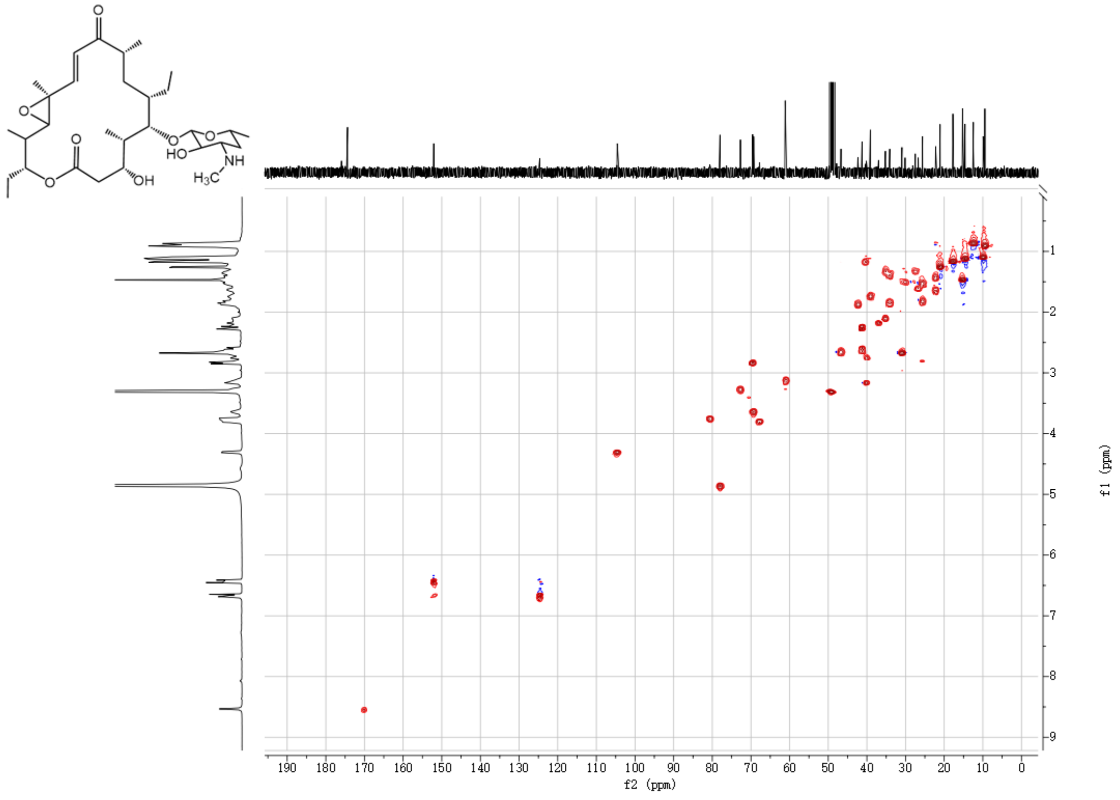

Figure S6 HSQC spectrum of compound **1** in CD_3OD

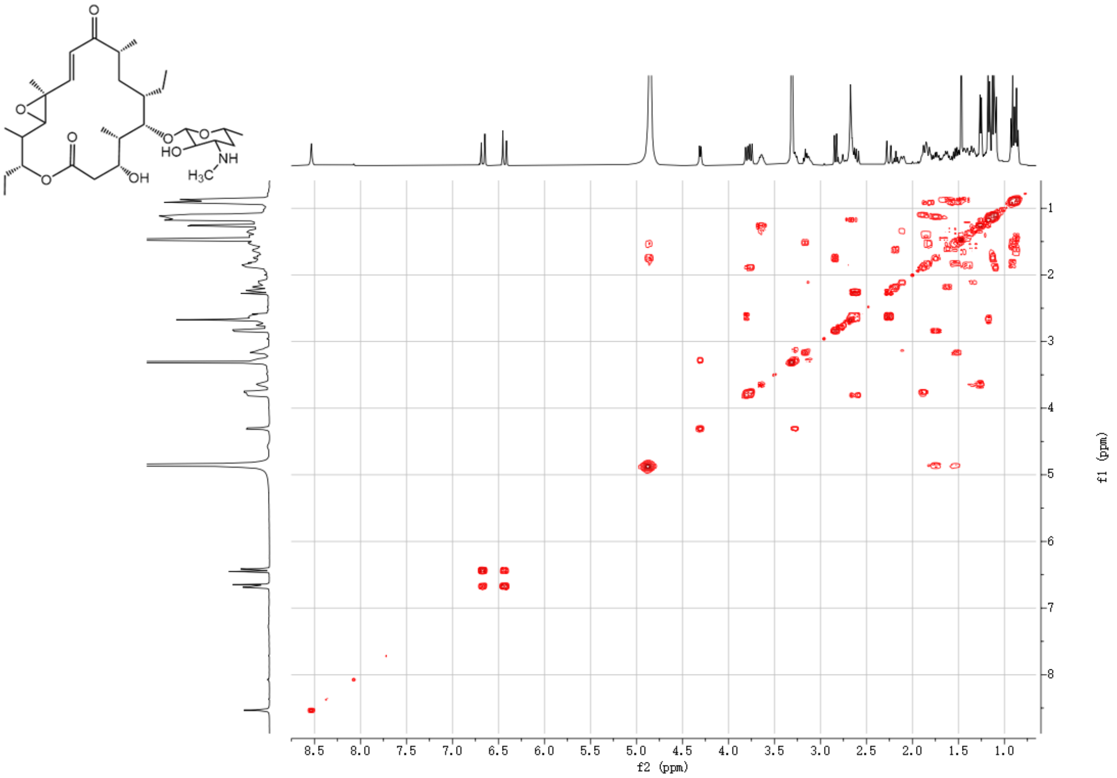

Figure S7 ^{1}H-^{1}H COSY spectrum of compound **1** in CD_3OD

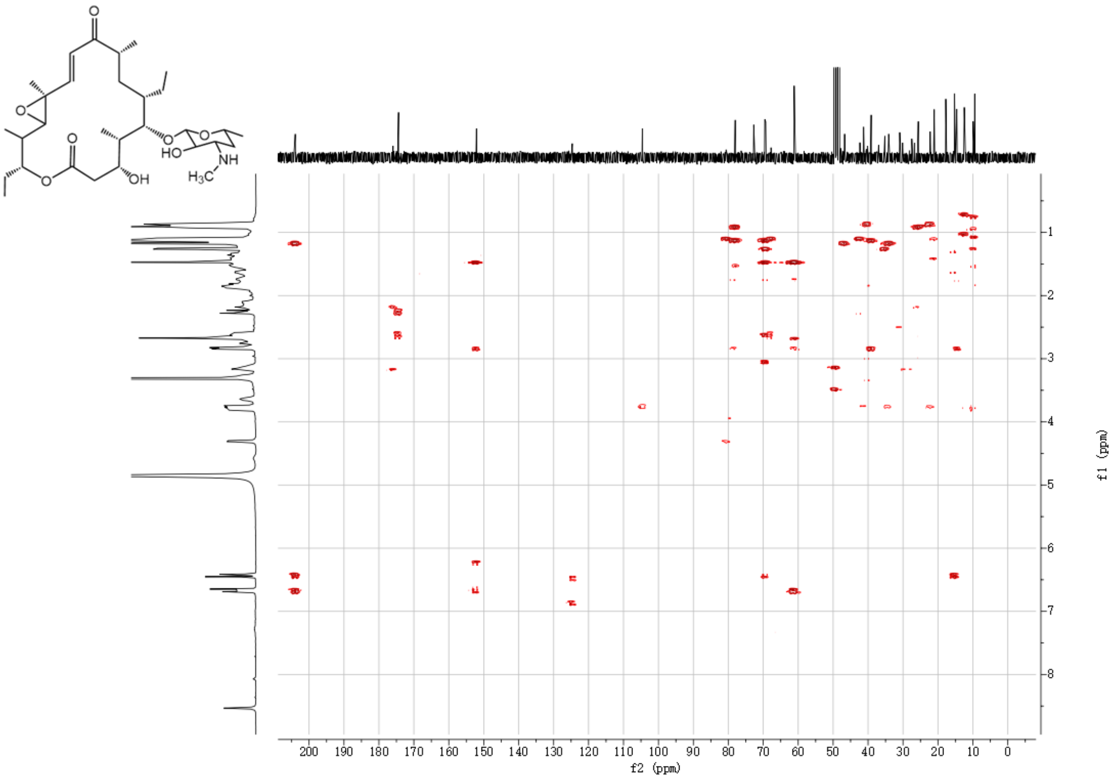

Figure S8 HMBC spectrum of compound **1** in CD_3OD

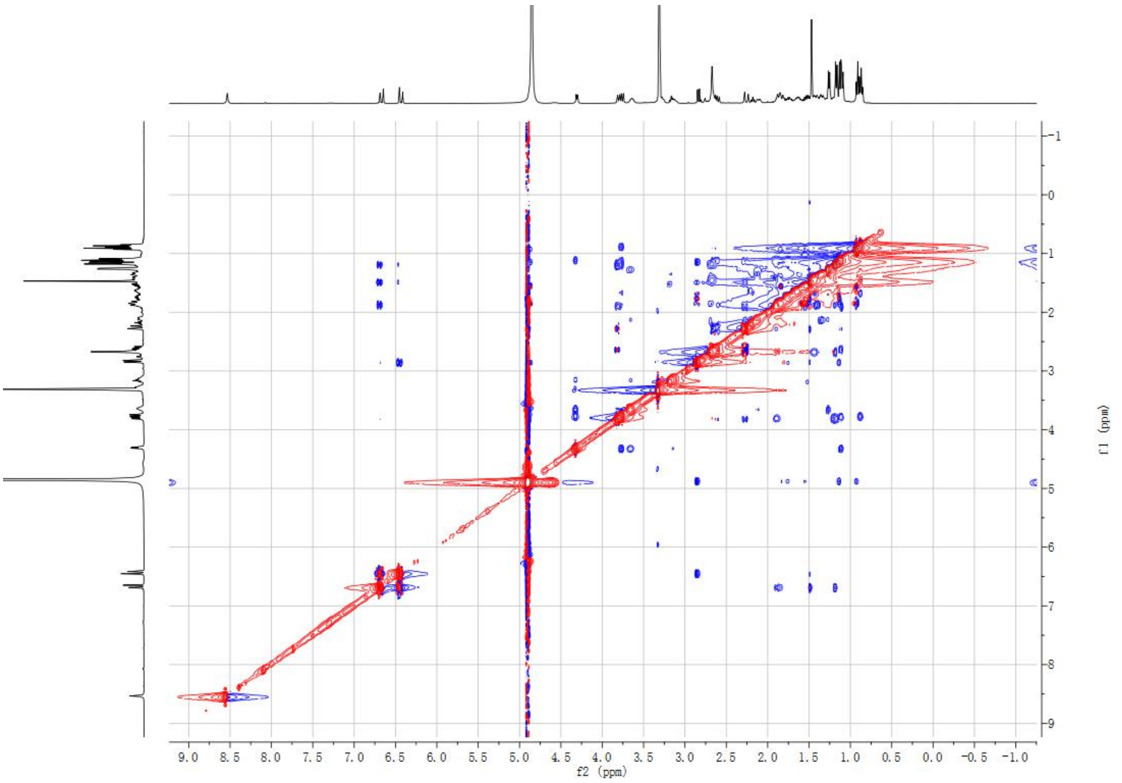

Figure S9 NOESY spectrum of compound **1** in CD_3OD

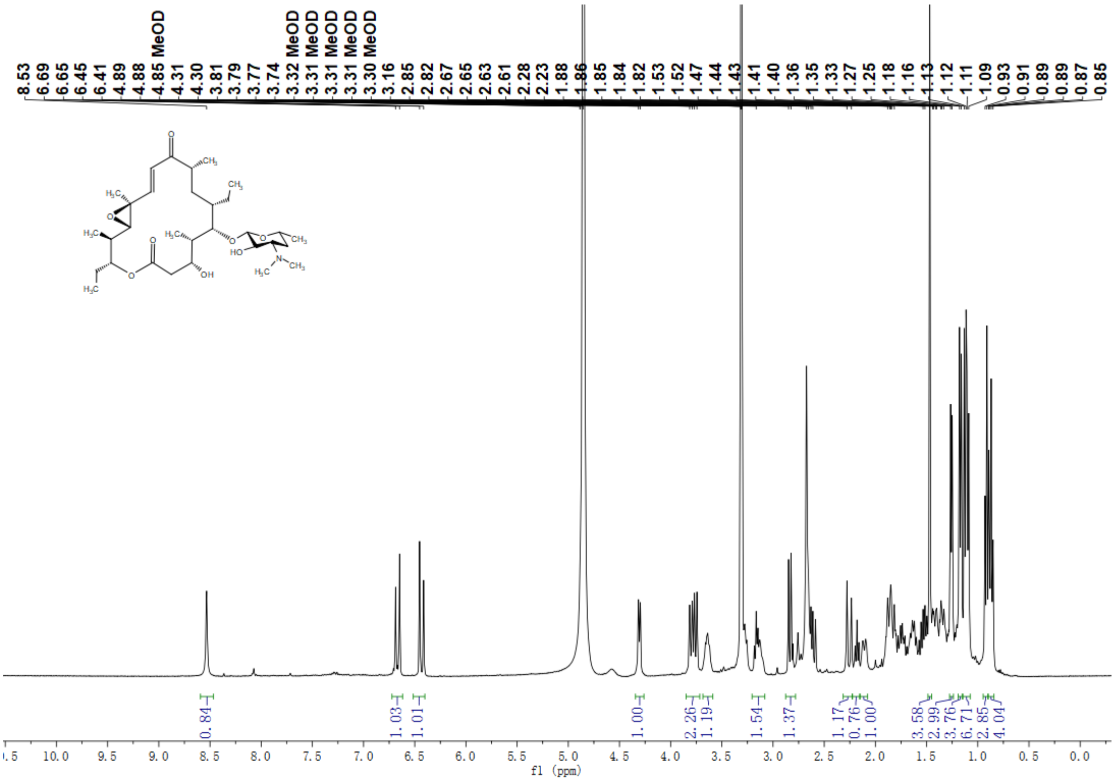

Figure S10 ^{1}H NMR (400 MHz) spectrum of compound **2** in CD_3OD

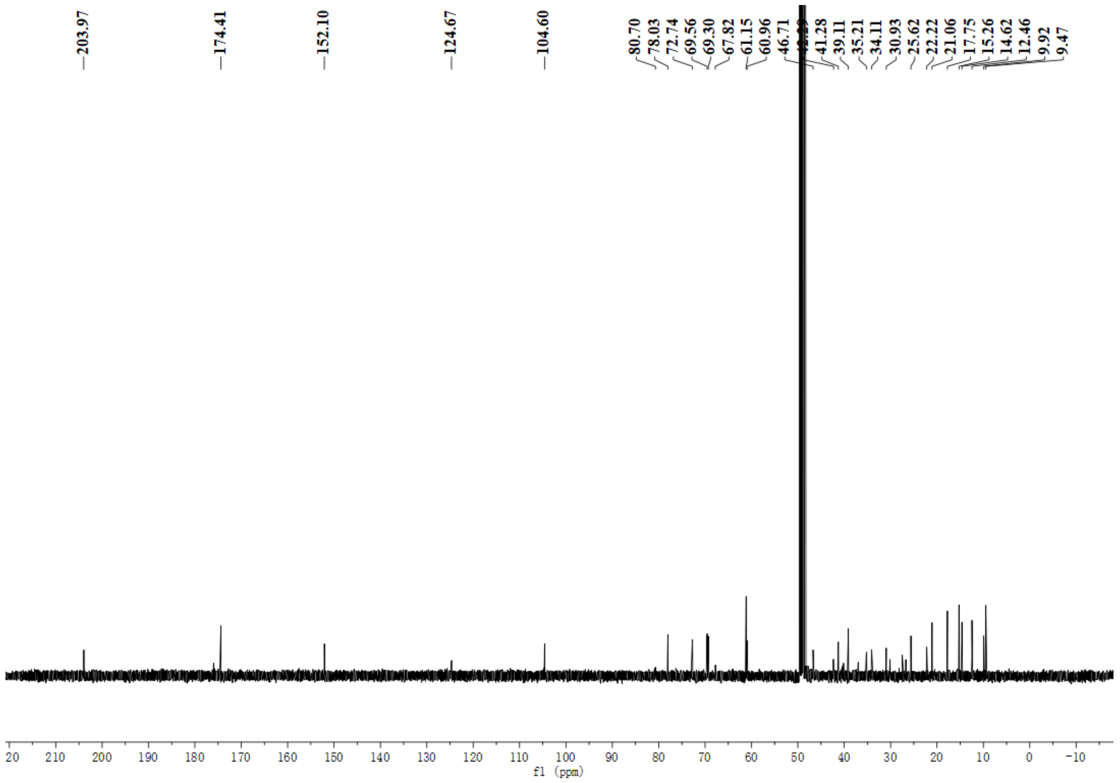

Figure S11 ^{13}C NMR (100 MHz) spectrum of compound **2** in CD_3OD

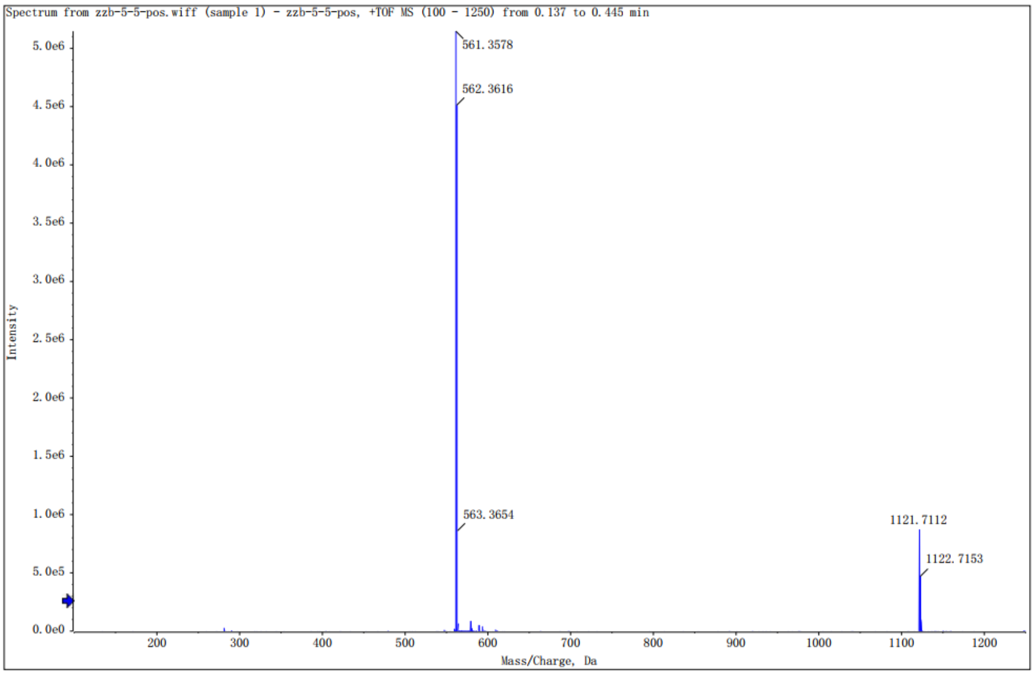

Figure S12 HRESIMS spectrum of compound **3**

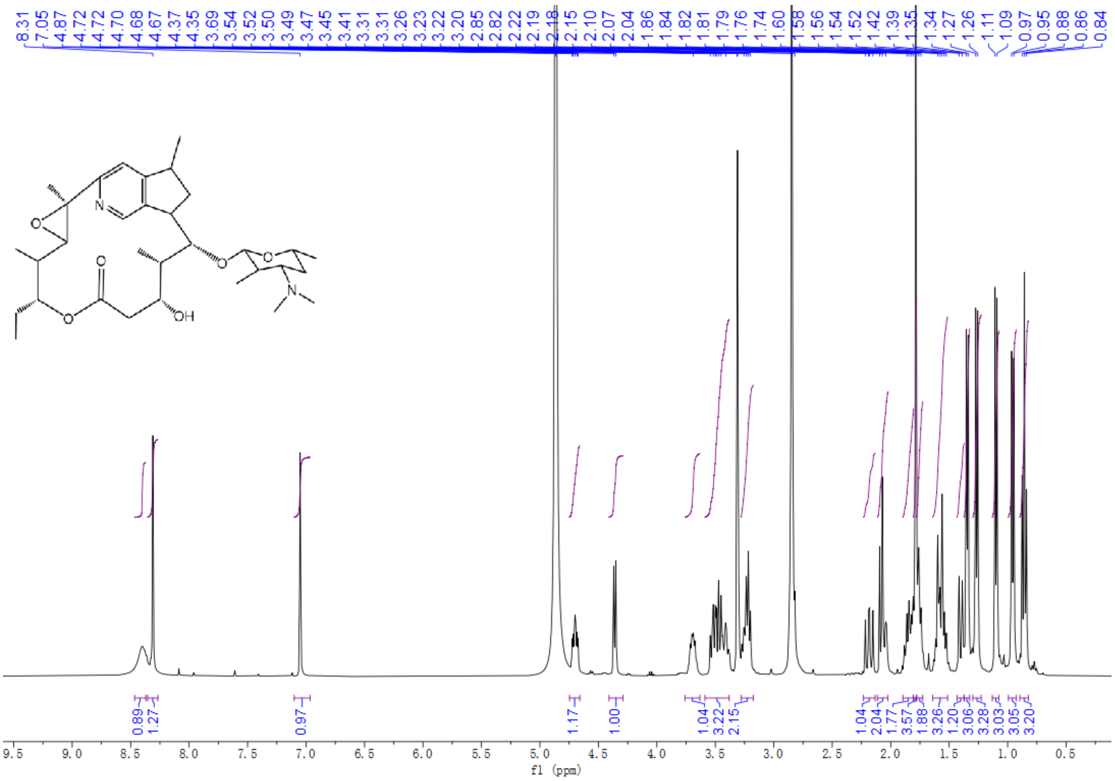

Figure S13 ^{1}H NMR (400 MHz) spectrum of compound **3** in CD_3OD

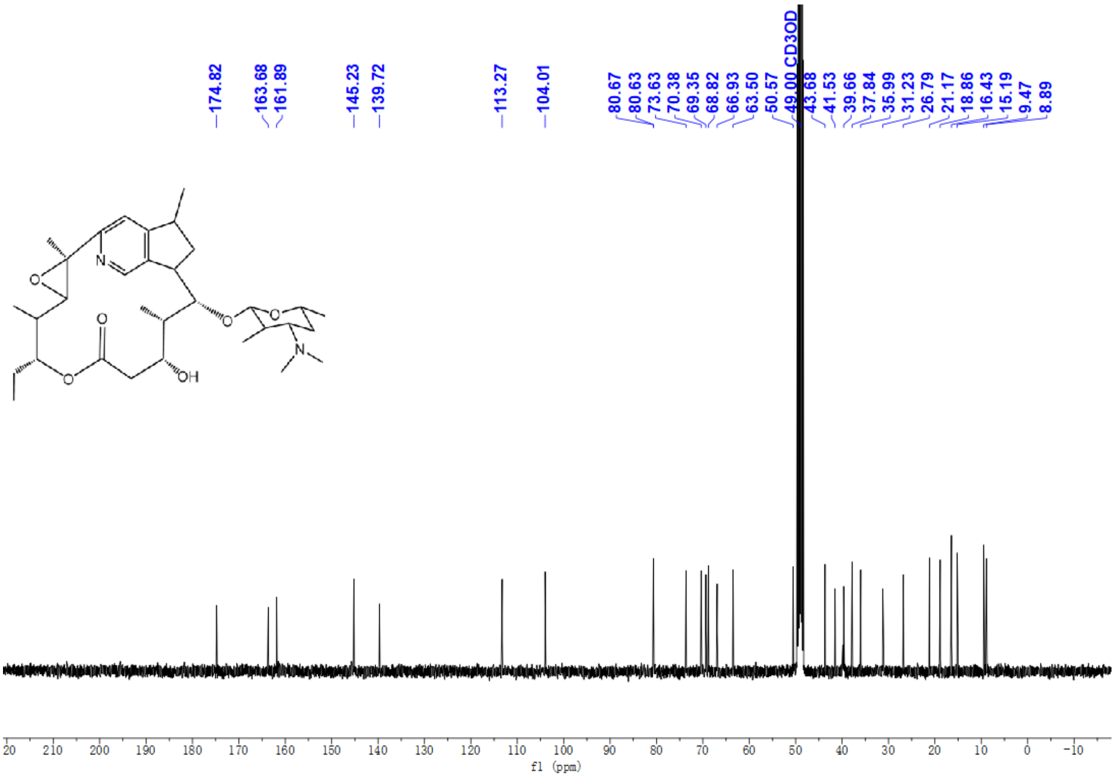

Figure S14 ^{13}C NMR (100 MHz) spectrum of compound **3** in CD_3OD

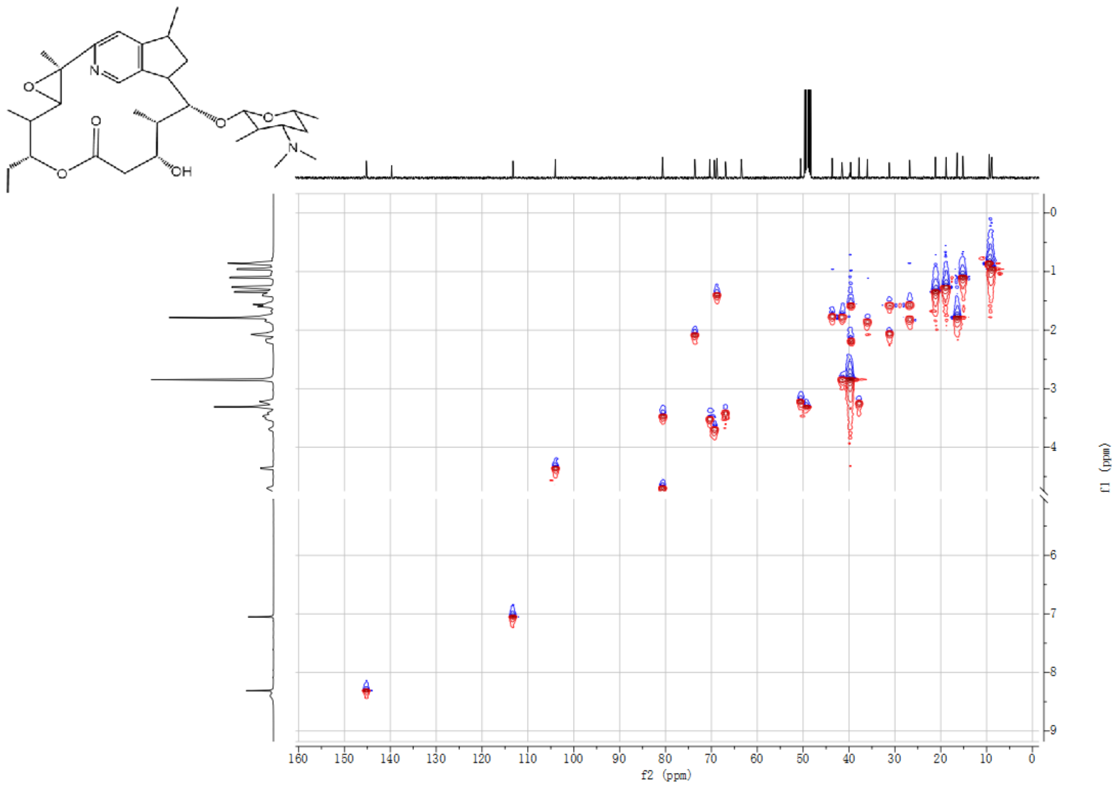

Figure S15 HSQC spectrum of compound **3** in CD_3OD

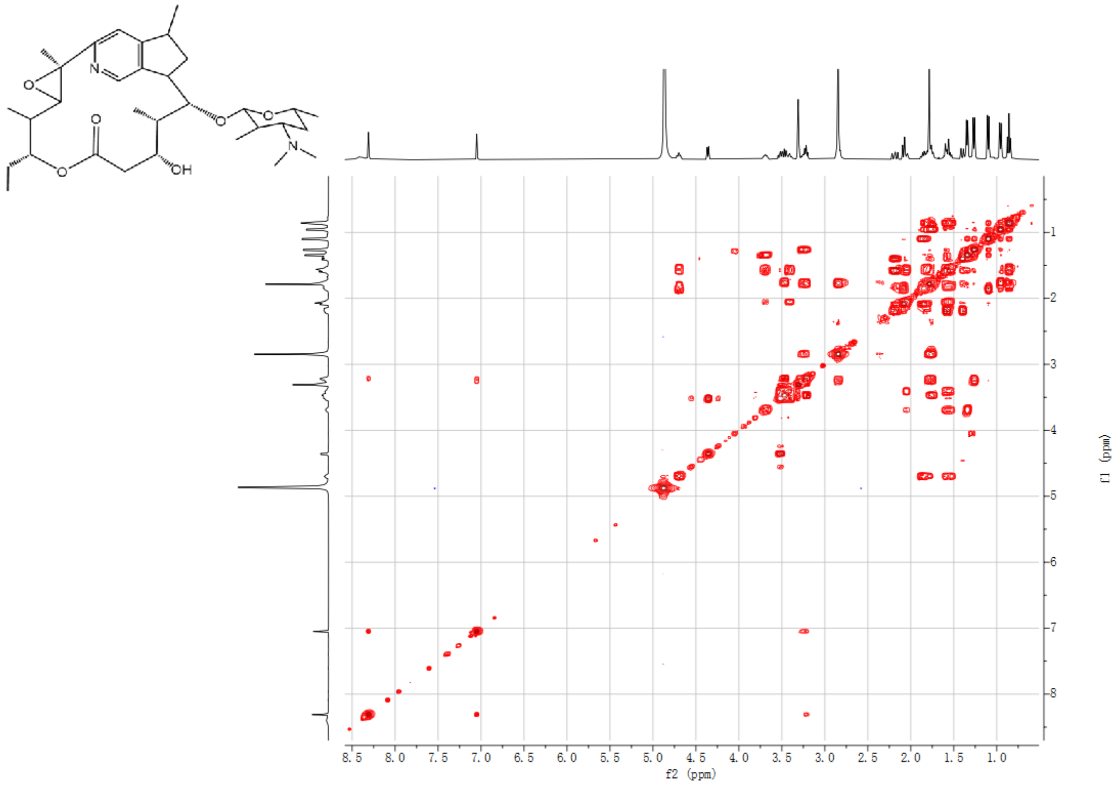

Figure S16 ^1H-^1H COSY spectrum of compound **3** in CD_3OD

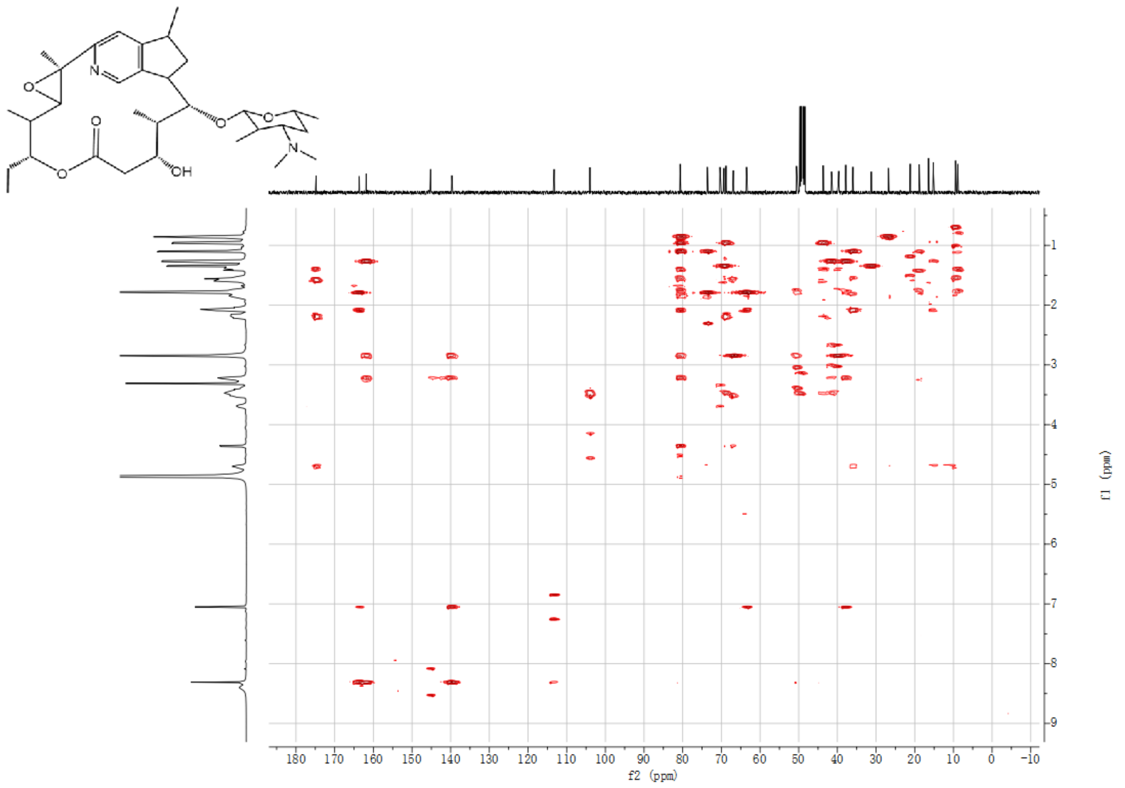

Figure S17 HMBC spectrum of compound **3** in CD_3OD

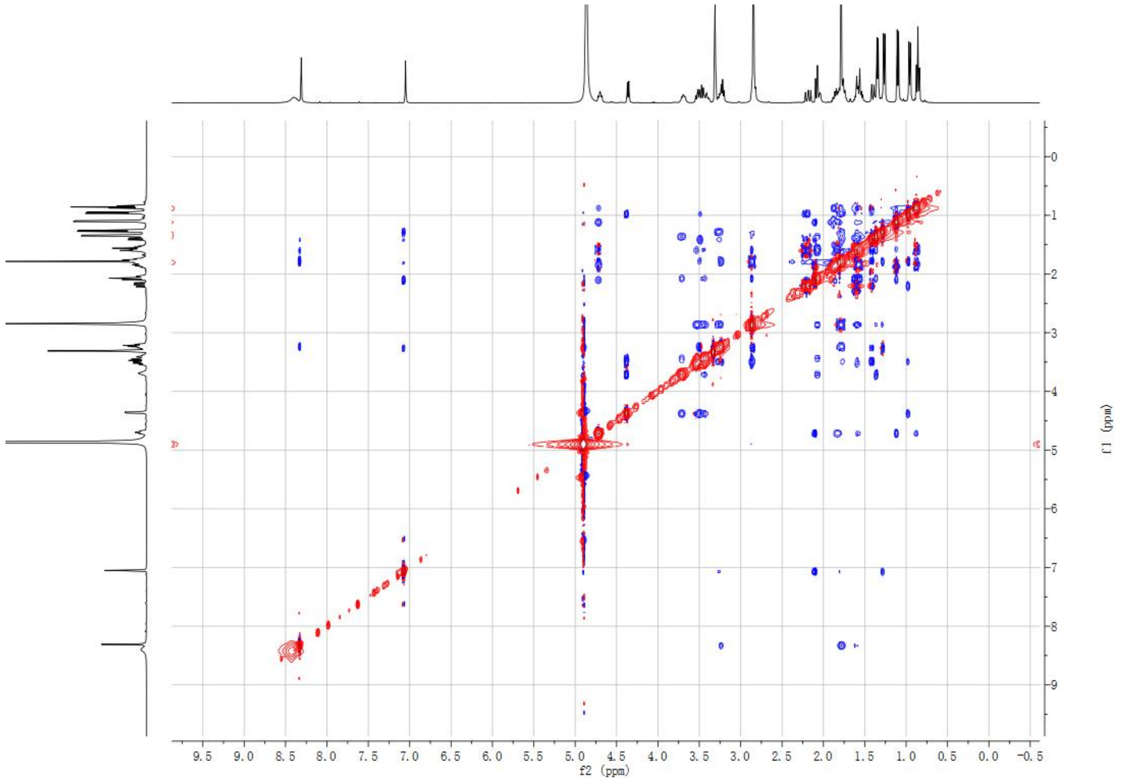

Figure S18 NOESY spectrum of compound **3** in CD_3OD

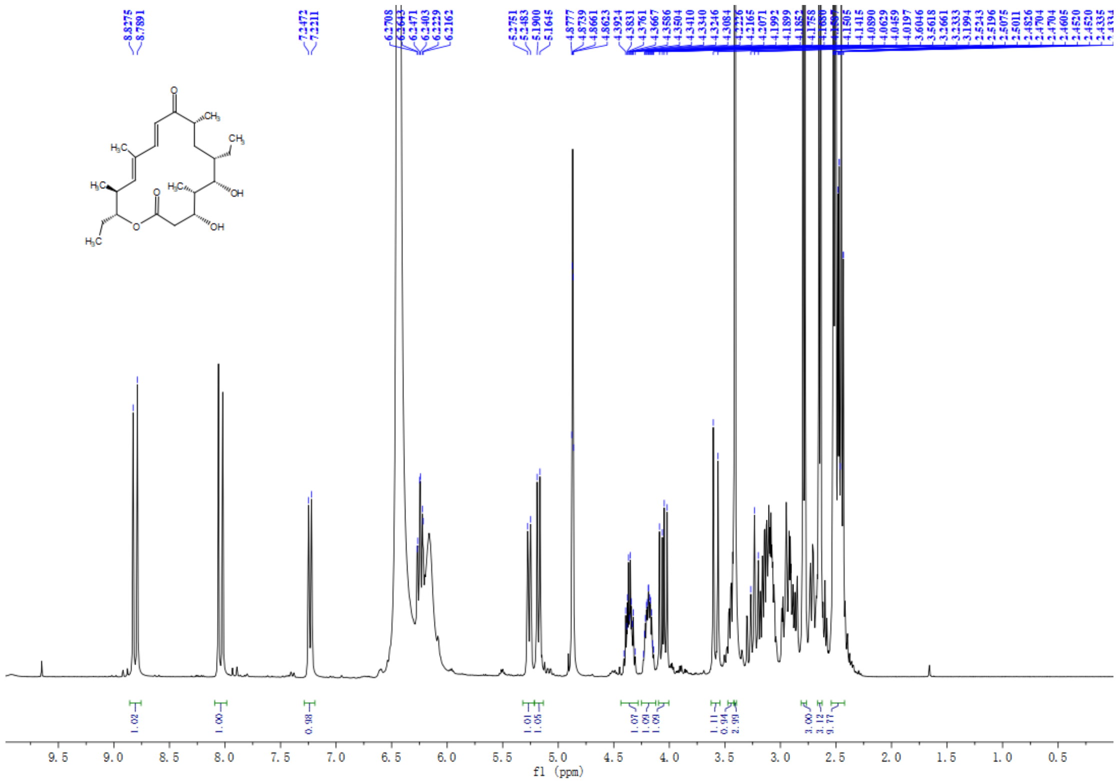

Figure S19 ^{1}H NMR(400 MHz) spectrum of compound **4** in CD_3OD

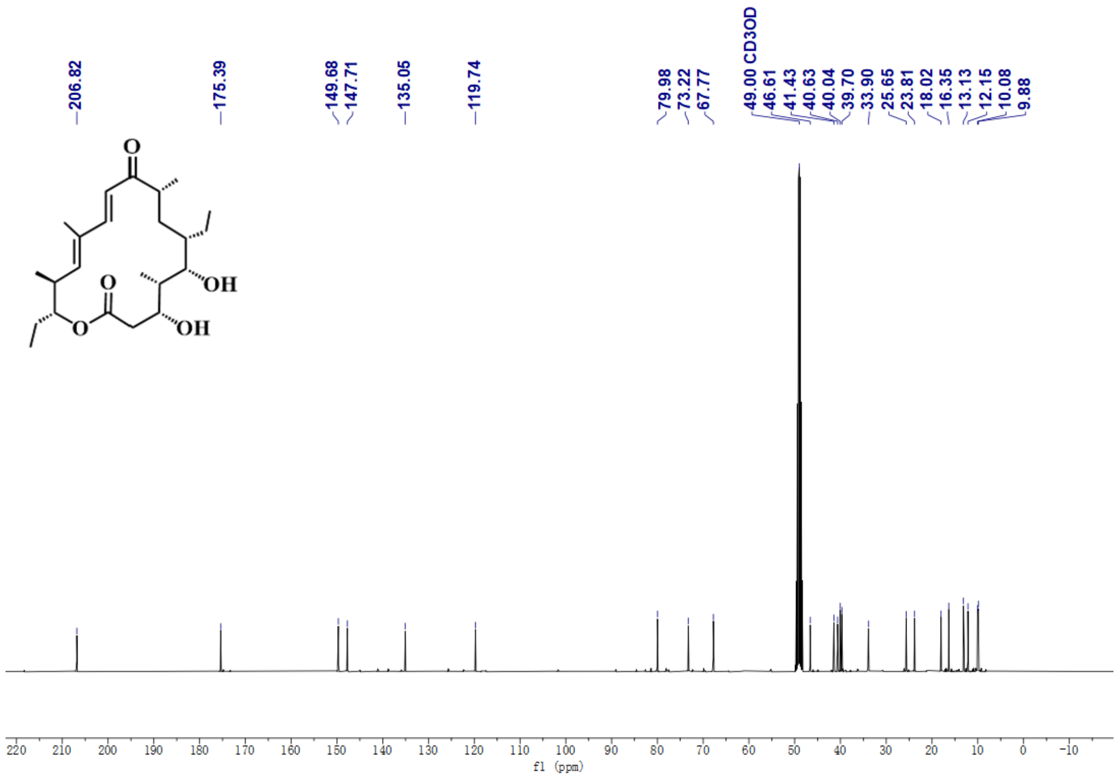

Figure S20 ^{1}C NMR (100 MHz) spectrum of compound **4** in CD_3OD

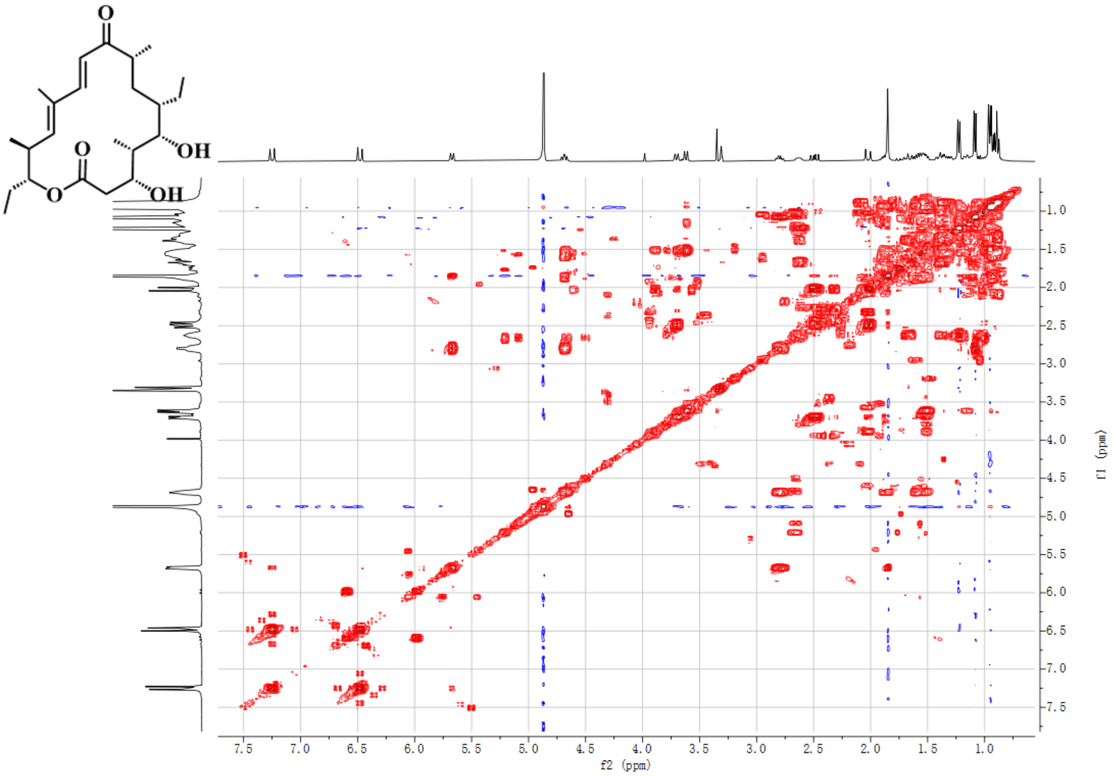

Figure S21 ^{1}H-^{1}H COSY spectrum of compound **4** in CD_3OD

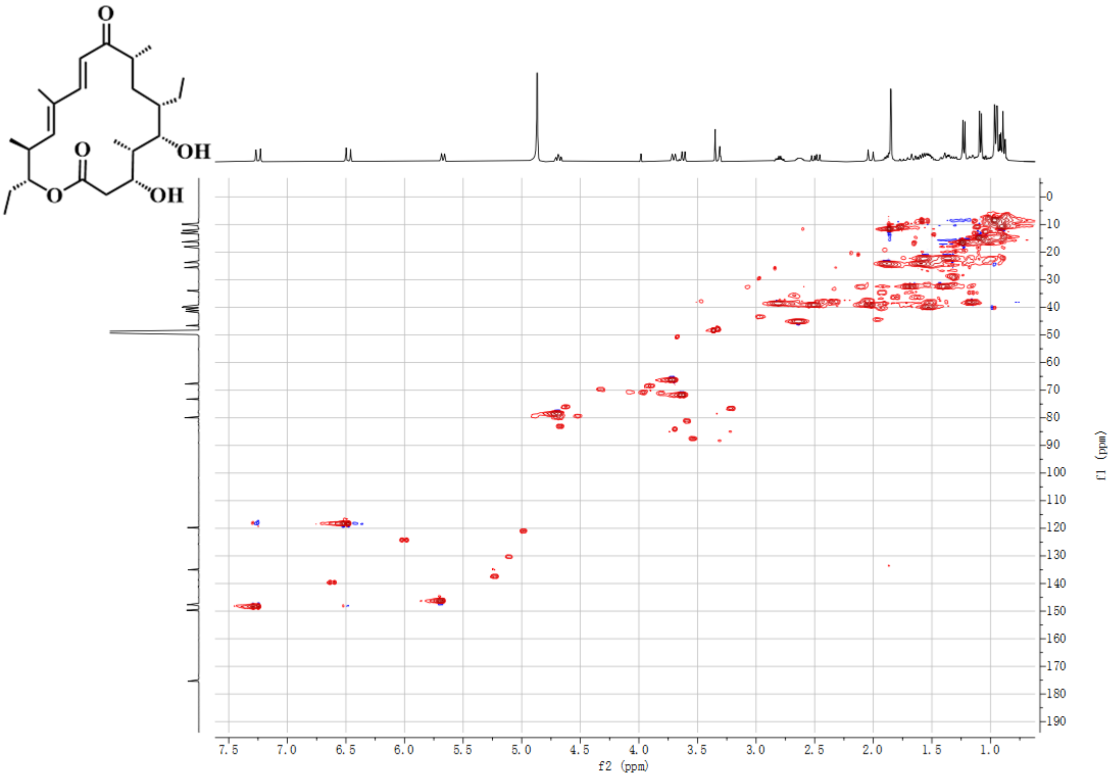

Figure S22 HSQC spectrum of compound **4** in CD_3OD

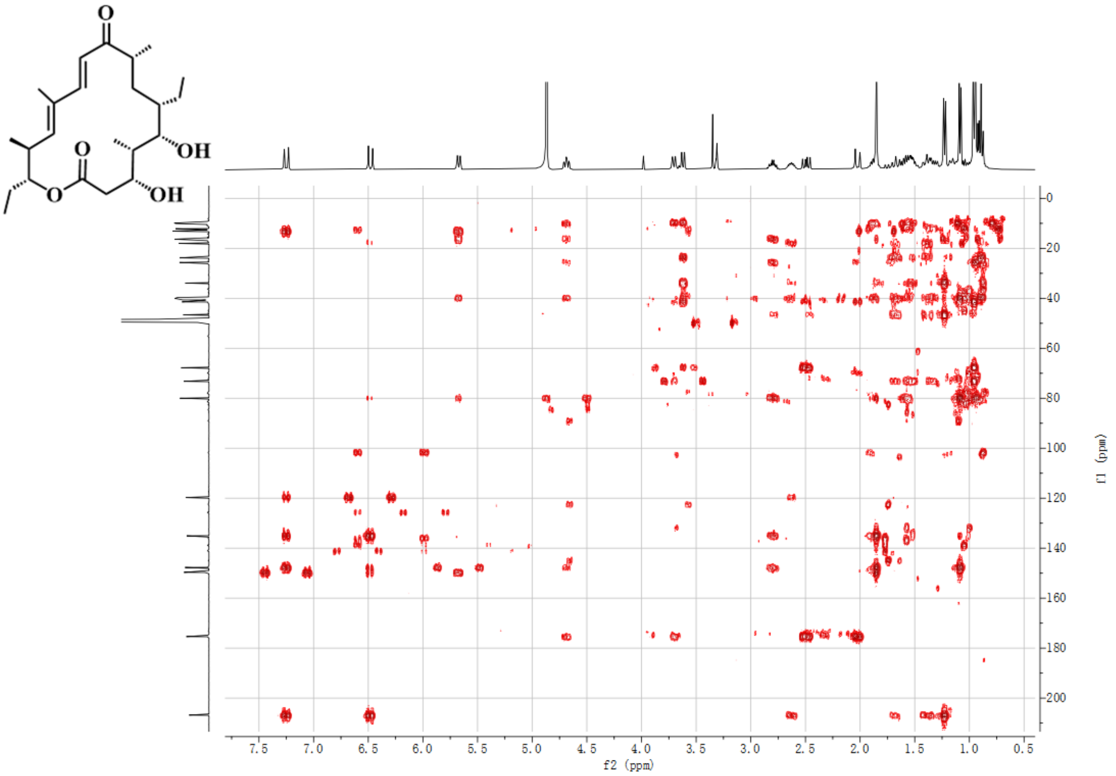

Figure S23 HMBC spectrum of compound **4** in CD_3OD

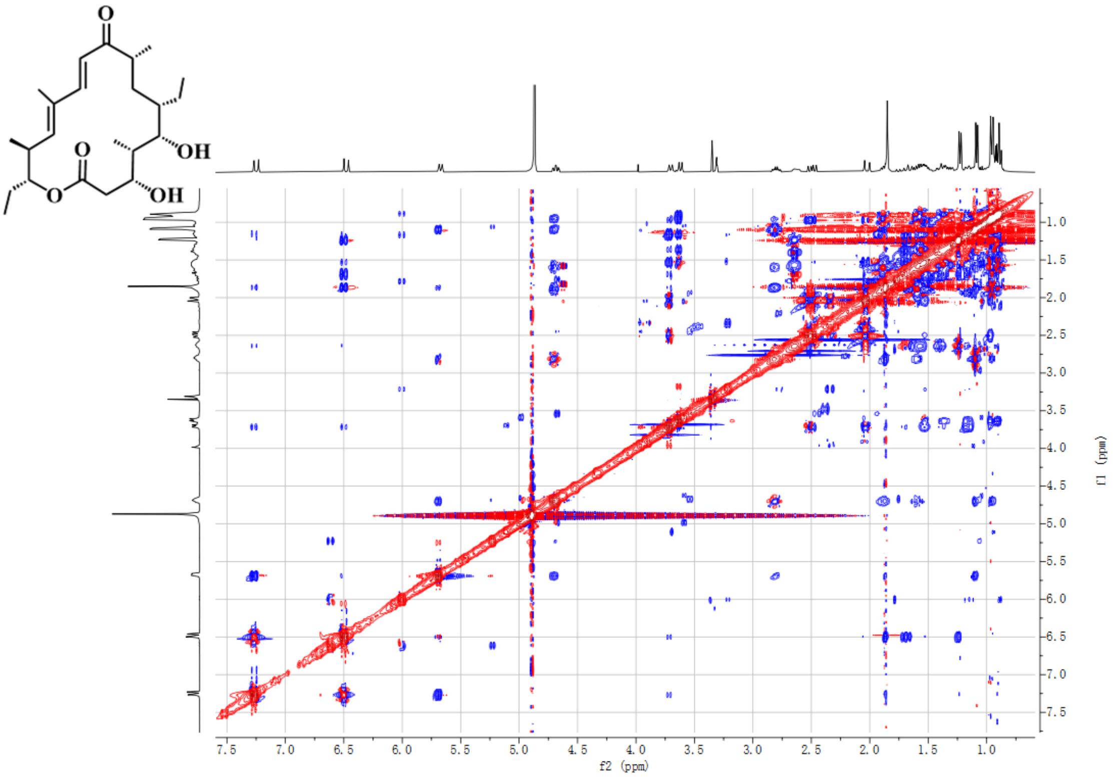

Figure S24 NOESY spectrum of compound **4** in CD_3OD

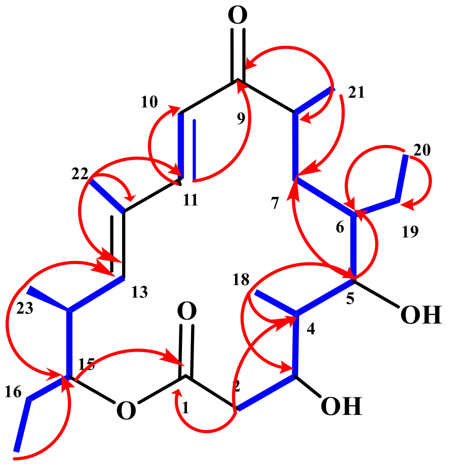

Fig. S25 The ^{1}H–^{1}H COSY (▬) and key HMBC (→) correlations of **4**

Table S1. Biosynthesis gene clusters for secondary metabolites in *Micromonospora* sp. FoRo54 predicted by antiSMASH

No.	Type	Predicted structure	Gene cluster homology
1	blactam	tabtoxin	18%
2	NRPS,T1PKS	thiocoraline	34%
3	phenazine	endophenazine A/B	44%
4	NRPS,betalactone	teleocidin B1	50%
5	NRPS,T1PKS	asukamycin	6%
6	lanthipeptide	-	-
7	PKS-like	rustmicin	33%
8	lanthipeptide,terpene	SapB	100%
9	T2PKS	xantholipin	14%
10	NRPS,lanthipeptide	RP-1776	8%
11	hglE-KS,T1PKS,T2PKS	fluostatin	100%
12	T1PKS	Rosamicin/salinipyrone A	91%
13	linaridin	-	-
14	NRPS	foxicins A-D	4%
15	NRPS,T1PKS	-	-
16	bacteriocin	Lymphostin/neolymphostinol B	30%
17	lipolanthine	kanamycin	1%
18	NRPS,T1PKS	JBIR-76/JBIR-77	8%
19	resorcinol	kijanimicin	4%
20	T3PKS	dihydrogeranyl-methoxyhydroquinones	71%
21	linaridin	Pentostatine/vidarabine	6%
22	Terpene	isorenieratene	25%
23	terpene	phosphonoglycans	3%
24	siderophore	desferrioxamine E	100%
25	T2PKS	FD-594	39%
26	T1PKS	sporolide A/sporolide B	21%
27	NAGGN	-	18%

Note:. NRPS: Nonribosomal peptide synthetase; PKS: Polyketide synthase; T1-3 PKS: Type I-III Polyketide synthase. “-”: Analysis of gene cluters did not yield specific secondary metabolitics

Table S2 Deduced functions of ORFs in the 12# biosynthetic gene cluster

ORF	Size	Proposed function	ID/SM	Protein homologue and origin
Ros1	230	LuxR family transcriptional regulator	70/80	(WP_127831713.1) *Streptomyces* sp. San01
Ros2	442	histidine kinase	57/67	(WP_158994189.1) *Streptomyces* sp. QHH-9511
Ros3	269	glucose-1-phosphate thymidylyltransferase RfbA	86/92	(WP_132399237.1) *Micromonospora* sp. KC207
Ros4	329	dTDP-glucose 4,6-dehydratase	88/92	(WP_229403228.1)*Micromonospora* sp. TP-A0468
Ros5	277	23S ribosomal RNA methyltransferase Erm	83/90	(WP_132399239.1)*Micromonospora* sp. KC207
Ros6	476	dTDP-4-amino-4,6-dideoxy-D-glucose ammonia-lyase	86/90	(WP_132399241.1)*Micromonospora* sp. KC207
Ros7	409	dTDP-4-dehydro-6-deoxyglucose aminotransferase	96/97	(WP_123606372.1)*Micromonospora* sp. Llam0
Ros8	466	GTPase HflX	95/96	(WP_199758113.1)*Micromonospora* sp. Llam0
Ros9	670	helix-turn-helix domain-containing protein	94/96	(WP_123606375.1)*Micromonospora* sp. Llam0
Ros10	452	crotonyl-CoA carboxylase/reductase	97/99	(ROO52172.1)*Micromonospora* sp. Llam0
Ros11	238	class I SAM-dependent methyltransferase	98/98	(WP_123606377.1)*Micromonospora* sp. Llam0
Ros12	431	activator-dependent family glycosyltransferase	96/98	(WP_123606378.1)*Micromonospora* sp. Llam0
Ros13	434	P450-derived glycosyltransferase activator	93/94	(WP_148083760.1)*Micromonospora* sp. Llam0
Ros14	1811	tylactone synthase	93/94	(ROO52176.1)*Micromonospora* sp. Llam0
Ros15	1575	tylactone synthase/type I polyketide synthase PikAIII	93/94	(ROO52177.1)*Micromonospora* sp. Llam0
Ros16	3495	type I PKS module 4, module 5	78/83	(ARW71485.1)*Micromonospora chalcea* subsp. izumensis
Ros17	1873	type I polyketide synthase	90/92	(WP_123606382.1)*Micromonospora* sp. Llam0
Ros18	3790	type I PKS loading module, module 1, module 2	73/80	(ARW71483.1)*Micromonospora chalcea* subsp. izumensis
Ros19	575	type I polyketide synthase	88/89	(WP_123606383.1)*Micromonospora* sp. Llam0
Ros20	403	cytochrome P450	95/97	(WP_123606384.1)*Micromonospora* sp. Llam0
Ros21	405	cytochrome P450	96/97	(WP_123606385.1)*Micromonospora* sp. Llam0
Ros22	381	DegT/DnrJ/EryC1/StrS family aminotransferase	94/96	(WP_123606386.1)*Micromonospora* sp. Llam0
Ros23	257	alpha/beta fold hydrolase	92/95	(WP_123606387.1)*Micromonospora* sp. Llam0
Ros24	551	transcriptional regulator	95/96	(WP_123606388.1)*Micromonospora* sp. Llam0
Ros25	189	nucleosidase	72/83	(WP_169597355.1)Micromonospora sp. HNM0581
Ros26	314	acyltransferase domain-containing protein	87/94	(WP_079313821.1)Microbispora sp. GKU 823
Ros27	1096	transcriptional regulator	96/96	(RUL92184.1)Verrucosispora sp. FIM060022
Ros28	547	monooxygenase	99/99	(WP_428964984.1) Micromonospora sp. JCM 30529
Ros29	137	hypothetical protein	98/100	(WP_428964979.1)Micromonospora sp. JCM 30529

Ros30	509	MFS transporter	98/98	(WP_428964978.10) Micromonospora sp. JCM 30529
Ros31	199	TetR family transcriptional regulator	100/100	(WP_428964977.1) Micromonospora sp. JCM 30529
Ros32	329	substrate-binding domain-containing protein	98/98	(WP_421433785.1) Solwaraspora sp. WMMB335

Size: Numbers indicate amino acids; ID:identity; SM: similarity.

Table S3 ^{1}H NMR (400 MHz) and ^{13}C NMR (100 MHz) data for **3** and **4** in CD_3OD

No.	**3**		**4**	
	δ_C, type	δ_H mult (J in Hz)	δ_C, type	δ_H mult (J in Hz)
1	174.8, C		175.4, C	
2	39.7, CH_2	1.57 dd (14.5 7.0)<br>2.20 dd (14.5 11.4)	40.7, CH_2	2.49 dd (17.3 10.5)<br>2.02 d (17.2)
3	68.8. CH	3.46 d (11.4)	67.8, CH	3.70 d (10.7)
4	43.7, CH	1.78 m	41.4, CH	1.83-1.90 m
5	80.6, CH	3.49 t (8.1)	73.2, CH	3.62 d (10.2)
6	50.6, CH	3.24 t (7.0)	39.7, CH	1.16-1.21 m
7	41.5, CH_2	1.80 m<br>2.86 m	33.9, CH_2	1.42 m<br>1.67 br t
8	37.8, CH	3.27 m	46.6, CH	2.63-2.69 m
9	161.9, C		206.9, C	
10	113.3, CH	7.07 s	119.7, CH	6.48 d (15.4)
11	163.7, C		149.7, CH	7.25 d (15.4)
12	63.5, C		135.0, C	
13	73.6, CH	2.10 d (9.4)	147.8, CH	5.67 d (10.5)
14	36.0, CH	1.82-1.91 m	40.0, CH	2.80 tq (10.2 6.5)
15	80.7, CH	4.72 ddd (9.5 7.7 3.5)	80.0, CH	4.68 td (9.6 2.6)
16	26.8, CH_2	1.52-1.63 m<br>1.78-1.90 m	25.6, CH_2	1.58 m<br>1.88 m
17	9.5, CH_3	0.88 t (7.4)	10.1, CH_3	0.94 t (7.4)
18	8.9, CH_3	0.98 d (7.2)	9.9, CH_3	0.95 d (6.7)
19	139.7, C		23.8, CH_2	1.36 m<br>1.55 m
20	145.2, CH	8.33 s	12.2, CH_3	0.89 t (7.4)
21	18.9, CH_3	1.28 d (6.9)	18.0, CH_3	1.23 d (6.9)
22	16.4, CH_3	1.80 s	13.1, CH_3	1.85 s
23	15.2,CH_3	1.12 d (6.7)	16.3, CH_3	1.09 d (6.5)
1'	104.0, CH	4.38 d (7.1)		
2'	70.4, CH	3.54 dd (10.4 7.3)		
3'	66.9, CH	3.42 m		
4'	31.2, CH_2	2.08 br d (12.2)<br>1.60 m		
5'	69.3, CH	3.71 m		
6'	21.2, CH_3	1.36 d (6.1)		
7'	39.8, CH_3	2.86 s		
8'	39.8, CH_3	2.86 s		
